# Boron Stress Responsive MicroRNAs and Their Targets in Barley

**DOI:** 10.1371/journal.pone.0059543

**Published:** 2013-03-26

**Authors:** Esma Ozhuner, Vahap Eldem, Arif Ipek, Sezer Okay, Serdal Sakcali, Baohong Zhang, Hatice Boke, Turgay Unver

**Affiliations:** 1 Department of Biology, Faculty of Science, Cankiri Karatekin University, Cankiri, Turkey; 2 Department of Biology, Faculty of Science, Istanbul University, Istanbul, Turkey; 3 Department of Biology, Faculty of Arts and Sciences, Suleyman Demirel University, Isparta, Turkey; 4 Department of Biology, East Carolina University, Greenville, North Carolina, United States of America; Nanjing Agricultural University, China

## Abstract

Boron stress is an environmental factor affecting plant development and production. Recently, microRNAs (miRNAs) have been found to be involved in several plant processes such as growth regulation and stress responses. In this study, miRNAs associated with boron stress were identified and characterized in barley. miRNA profiles were also comparatively analyzed between root and leave samples. A total of 31 known and 3 new miRNAs were identified in barley; 25 of them were found to respond to boron treatment. Several miRNAs were expressed in a tissue specific manner; for example, miR156d, miR171a, miR397, and miR444a were only detected in leaves. Additionally, a total of 934 barley transcripts were found to be specifically targeted and degraded by miRNAs. *In silico* analysis of miRNA target genes demonstrated that many miRNA targets are conserved transcription factors such as Squamosa promoter-binding protein, Auxin response factor (ARF), and the MYB transcription factor family. A majority of these targets were responsible for plant growth and response to environmental changes. We also propose that some of the miRNAs in barley such as miRNA408 might play critical roles against boron exposure. In conclusion, barley may use several pathways and cellular processes targeted by miRNAs to cope with boron stress.

## Introduction

MicroRNAs (miRNAs) are a class of single strand, endogenous, non-coding small RNA molecules, which post-transcriptionally regulate gene expression in many organisms by targeting mRNAs for cleavage or translation suppression [1], [2], [3]. Increasing evidence demonstrates that miRNAs play an important role in many biological and metabolic processesincluding regulation of plant growth, development and response to biotic and abiotic stresses via interactions with their specific target mRNAs [4], [5], [6], [7], [8]. Boron (B) is an essential element for plants, and its deficiency generally causes growth defects mainly in young and growing parts of the plants, while excessive levels of B are toxic to plants [9], [10]. A number of physiological processes are shown to be altered by B exposure. Deterioration of cell wall biosynthesis, metabolic deterioration by binding to the ribose moieties of ATP, NADH and NADPH, and inhibition of cell division and elongation are the most distinct symptoms of B toxicity [11], [12], [13]. However, plants also evolve mechanims to cope with the presence of excessive amounts of metal ion. Although several studies have been performed on small RNAs and metal stressors such as mercury, cadmium, and aluminum [14], [15], [16], no studies have been reported on boron stress.

Barley (*Hordeum vulgare* L.) is one of the most important grain crops grown and cultivated worldwidely [17]. Additionally, it is a well-studied model plant for triticacea research in terms of genetics, genomics, and breeding [18], [19]. Although miRNAs in barley were identified in previous studies [19], [20], [21], [22], compared with the number of identified miRNAs in other grain crops such as rice and maize, the number of known miRNAs in barley is still very insufficient. Initially, conventional approaches were extensively used for miRNA identification and contributed considerably to the miRNA exploration [8], [23].

The purpose of this study is to identify tissue specific expression of miRNAs and their potential targets in barley exposed to high levels of boron. To achieve this goal, we identified miRNAs from the entire transcriptome RNA-seq data, which included more than 208 million reads generated from control and B-exposed roots and leaves of B-tolerant barley seedlings. Some of the identified barley miRNAs were validated in leaf and root tissues by quantitative RT-PCR. Additionally, ‘degradome sequencing’ approach was also employed for miRNA target identification in barley.

## Materials and Methods

### Plant Materials and Boron Treatment

Barley (*Hordeum vulgare* L. cultivar Sahara) seeds were sterilized and placed into Petri dishes for germination at room temperature. Then, four-day-old seedlings were transferred into liquid culture flasks including nutrient solutions. The treatments were repeated at least three times with triple biological replicates. For toxicity experimets, toxic (1000 µM) and nontoxic (50 µM) concentrations of B were added to different flasks. Germinated seedlings were exposed to B-toxic or B-nontoxic conditions for approximately 24 hours.

### RNA Isolation, cDNA Library Construction and Sequencing for Transcriptome Analysis

Total RNAs were extracted from barley root and leaf tissues using the TRIZOL Reagent (Invitrogen) according to the manufacturer’s instructions. The extractions were performed separately for each sample with three independent biological replicates and same amount of total RNA was subsequently pooled based on their concentration. The quality and quantity of purified RNAs were assessed with a Nanodrop 2000c spectrophotometer (Nanodrop Technologies, USA) and the presence of ribosomal RNA bands was determined by Agilent 2100 Bioanalyzer (Agilent Technologies, Santa Clara, CA). All RNA samples were stored at −80°C until further processing. The cDNA library construction and Illumina (Solexa) based-transcriptome sequencing experiments were conducted by the BGI (Beijing Genomics Institute, Hong Kong). In brief, for each library, the polyadenylated RNA (mRNAs) was isolated from 20 µg of each RNA pool using oligo(dT) 25 magnetic beads (Invitrogen) according to the manufacturer’s protocol. Following purification step, the isolated mRNAs were fragmented into small pieces using fragmentation buffer. These mRNA fragments resulted were used as templetes for first strand cDNA synthesis by reverse transcriptase and random hexamer primers. After completion of first-strand synthesis, the second-strand cDNA was synthesized by using DNA polymerase I, RNaseH, dNTPs and buffer. Subsequently, newly synthesized short fragments were purified with a QIAquick PCR extraction kit and further subjected to end reparation and adding poly(A) using EB buffer, T4 DNA polymerase, the Klenow fragment, and T4 polynucleotide as well as Klenow 3′ to 5′ exo-polymerase and then, the sequencing adapters were connected with those short fragments. After size selection and purification through agarose gel electrophoresis, the selected fragments were enriched by PCR amplification with appropriate primers and eventually, the library sequencing experiments were performed using Illumina HiSeq™ 2000 instrument.

### *De novo* Assembly of Transcriptome and Data Processing

Firstly, the image data obtained from sequencing platform were converted by base calling into sequence data which is commonly known as raw reads and typically stored in the fastaq file format. In order to acquire high-quality clear reads, all raw sequence reads were filtered to remove adaptors, the reads containing unknown nucleotides larger than 5% and low quality reads. Then, remaining clear reads were subjected to *de novo* transcriptome assembly using Trinity assembler with default settings [24]. Briefly, Inchworm, one of software module of Trinity, assembles reads with definite length of overlap in order to generate longer fragments which are termed contigs. Then, the reads are are realigned to the newly formed contig so as to construct scaffolds which are basically derived from the contigs from the same transcript. By using paired-end information, these reads (pair-end clean reads) are mapped back to the resultant scaffolds to fill the intra-scaffolds gaps. Subsequently, the sequences generated by assembly of scaffolds are defined as unigenes which cannot be extended on either end by further assembly process. After obtaining non-redundant unigenes as long as possible using sequence clustering software, some of those unigenes were termed by singletons which are are not part of any contigs.

### Identification of miRNAs and Prediction of miRNA Precursors (Pre-miRNAs)

The RNA-seq generated more than 208 million clean reads (Kekec et al. Unpublished data) that were used to identify miRNAs and their targets. To identify potential miRNA precursors (pre-miRNAs), Blastn search was perfromed with an e-value of 1e-10 between unigene reads constructed from a total of four libraries for whole transcriptome of *H. vulgare* and previously known pre-miRNA sequences obtained from miRBase v19.0 (http://www.mirbase.org/) and plant miRNA database (PMRD, http://bioinformatics.cau.edu.cn/PMRD/), respectively. Briefly, we created our own blast-search database from a fasta sequence file including all transcripts of *H. vulgare* to be used with Blastn algoritm. As a query, all plant pre-miRNAs were used to search against the database generated via Blastall-2.2.22. The output file was manually investigated to extract the longest possible set of matches from the batch sequences. Then, the chosen potential pre-miRNA sequences obtained from the result of Blastall were subjected to the Zuker folding algorithm for *in silico* secondary structure generation via the web-based computational software MFOLD 3.2 [25]. The default parameters of the software were adjusted for predicting secondary structure of the selected sequences and the minimal folding free energy index (MFEI) was calculated for each pre-miRNA sequence as described previously [26]. After identification of putative pre-miRNAs, we determined the localization of predicted mature miRNAs on the their own pre-miRNAs by mapping these mature miRNAs to the pre-miRNAs using BLAST search algorithm with default parameters. To consider whether these sequences are potential miRNA and pre-miRNA candidates, the following empirical criteria was adopted: (i) a pre-miRNA sequence can fold into an appropriate stem-loop hairpin secondary structure which contains 19–24 nucleotides mature miRNA in lengths, (ii) the mature miRNAs should take place in one arm of the hairpin structure, (iii) the minimum length of pre-miRNA sequences should be 60 nucleotides, (iv) mismatch between candidate mature miRNAs and query mature miRNAs are allowed to be up to 3 nucleotides, (v) it is strongly emphasized that MFEI and negative minimum folding energy (MFE) of potential miRNA precursors are relatively higher than those of other types of RNAs, and MFEI was considered to be one of the most important criterion for distinguishing miRNAs from other types of RNAs, e.g. pre-miRNA with approximately >0.67 has been identified as more likely to be a miRNA [27], (vi) there is no large loop or break in the miRNA:miRNA*, and (vii) the miRNA has less than six mismatches with the opposite miRNA sequence (miRNA*) on the opposite arm [28], [29], [30].

### Computational Identification of miRNA Target Genes

Identification of miRNA-regulated gene targets is crucial for understanding miRNA functions. Therefore, the putative targets of *H. vulgare* miRNAs were identified by aligning the miRNAs with the high-quality unigene set obtained from the assembled transcripts and the singleton transcripts of barley *de novo* transcriptome libraries using the web-based psRNA Target Server (http://plantgrn.noble.org/psRNATarget/) with default parameters of user-submitted option. Alignment between Hvu-miRNA and its potential target(s) was evaluated by the parameters described by Zhang [31]. These computationally identified miRNA targets were further analyzed using BlastX searches with an e-value of 1e-10 against *Hordeum* EST sequences at NCBI database to identify putative gene homologs for confirmation.

### Gene Ontology (GO) Analysis of Potential miRNA Targets

In order to better understand the functional roles of miRNAs in barley, Blast2Go (B2G) software v2.3.1 was used to assign gene ontology (GO) annotations of predicted target genes [32]. First, all miRNA target transcripts were arranged in a text file (Fasta format) as an input data and uploaded to the program for BlastX searches with an e-value of 1e-06. The BLAST results included: sequence length, gene name, e-value, similarity, Hit-length, Align-length, GenBank and Uniprot accession number as well as Gene Ontology IDs belonging to each target sequences. Next, the output file (.dat file) obtained from BlastX analysis was used to retrieve GO terms associated with each blast hit and Gene Ontology annotations. Finally, all miRNA targets representing genes with known function were categorized by biological process, cellular component and molecular function according to the ontological definitions of the GO terms.

### miRNA Target Cleavage Product (Degradome) Analysis

In order to characterize the miRNA cleaved target library (degradome) of *H. vulgare*, we evaluated a dataset derived from the output generated by CleaveLand (v2.0) software (a pipeline for using degradome data to find cleaved small RNAs). The miRNA-directed cleavage site in the miRNA:mRNA alignment is represented by red arrow (Table S1).

### Stem-loop Reverse-transcription

Stem-loop RT primers for Hvu-MIR 156, Hvu-MIR 159, Hvu-MIR 164, Hvu-MIR 166, Hvu-MIR 168, Hvu-MIR 171, Hvu-MIR 395, Hvu-MIR 396, Hvu-MIR 414, Hvu-MIR 1120 and Hvu-MIR 5048 were designed according to Varkonyi-Gasic et al. [33] (Table S2). The miRNA stem-loop reverse transcription was carried out using 500 ng of total RNA samples of B-nontoxic (50 µM) and B-toxic (1000 µM) leaf and root samples (1 µL), 0.5 µL 10 mM dNTP mix, 1 µL stem-loop RT primer (1 µM) and 10.5 µL nuclease free water. Those components were also mixed separately for the different dilutions of total RNA stem-loop RT primer for cDNA synthesis and incubated for 5 min at 65°C, and then put into ice for 2 min. Thereafter, 4 µL first-strand buffer (5×), 2 µL 1 M DTT, 0.1 µL RNAseOUT (40 units/µL), and 0.25 µL SuperScript III (200 units/µL) were added to each tube. The RT reactions were fulfilled as 30 min at 16°C followed by 60 cycles of 30°C for 30 s, 42°C for 30 s and 50°C for 1 s. The control tubes included all components without RT primer (no RT or - RT) and RNA template (no RNA or - RNA).

### Quantitative Real-time PCR

To verify some of the predicted *H. vulgare* miRNAs experimentally, and to measure and compare the expression levels of the miRNAs in root and leaf tissues treated with boron, qRT-PCR was conducted using SYBR Green I Master Kit (Roche, Germany) on a LightCycler® 480 Real-Time PCR System (Roche, Germany). For qRT-PCR analysis,10 µL 2X Master mix, 0.1 µL (100 pmol) forward and 0.1 µL (100 pmol) reverse primers, 7.8 µL nuclease-free water and 2 µL RT stem-loop cDNA products were used. Forward primers were specifically designed for each individual miRNA, but 5′-GTGCAGGGTCCGAGGT-3′ was used as the universal reverse primer [33] (Table S3). The qRT-PCR conditions were as follows: initial denaturation at 95°C for 10 min, followed by 41 cycles at 95°C for 10 s, 55°C for 20 s, and 72°C for 10 s. The melting curves were adjusted as 95°C for 5 s and 55°C for 1 min and then cooled to 40°C for 30 s. All reactions were repeated three times [30]. For each conditions, the qRT-PCR experiments were run as biological triplicates and expression levels were normalized according to pervious studies [8], [19], [28], [29], [33], [34]. The relative fold change for each comparison was calculated by 2^∧^-ΔCt after normalization [33], [34]. Error bars were derived from the three experiments in triplicate and error bars represent standard deviation.

### Validation of Barley miRNA Target mRNAs by qRT-PCR

To verify the expression levels of identified 11 barley miRNAs, the mature miRNAs were measured via qRT-PCR. Relative expression levels of predicted barley miRNAs were compared in root and leaf tissues treated with excess boron. The expression profile of these miRNA targets was also measured using qRT-PCR and their specific PCR primers were listed in the Table S3. The reverse transcription reaction was performed with Transcriptor High Fidelity cDNA Synthesis Kit (Roche, Germany) according to the manufacturer’s protocol. The qRT-PCR experiment was carried out as reported previously [34], [35]. Briefly, 2 µL cDNA was amplified with 0.1 µL specific primers in a total volume of 18 µL using SYBR Green I Master (Roche) with LightCycler® 480 Real-Time PCR System. *18s rRNA* (GenBank ID: AF147501) amplified with forward: GTGACGGGTGACGGAGAATT and reverse: GACACTAATGCGCCCGGTAT primers were used as a reference gene with triple replicates [36], [37].

## Results

### Identification of Boron Responsive miRNAs in Barley

**Figure 1:**
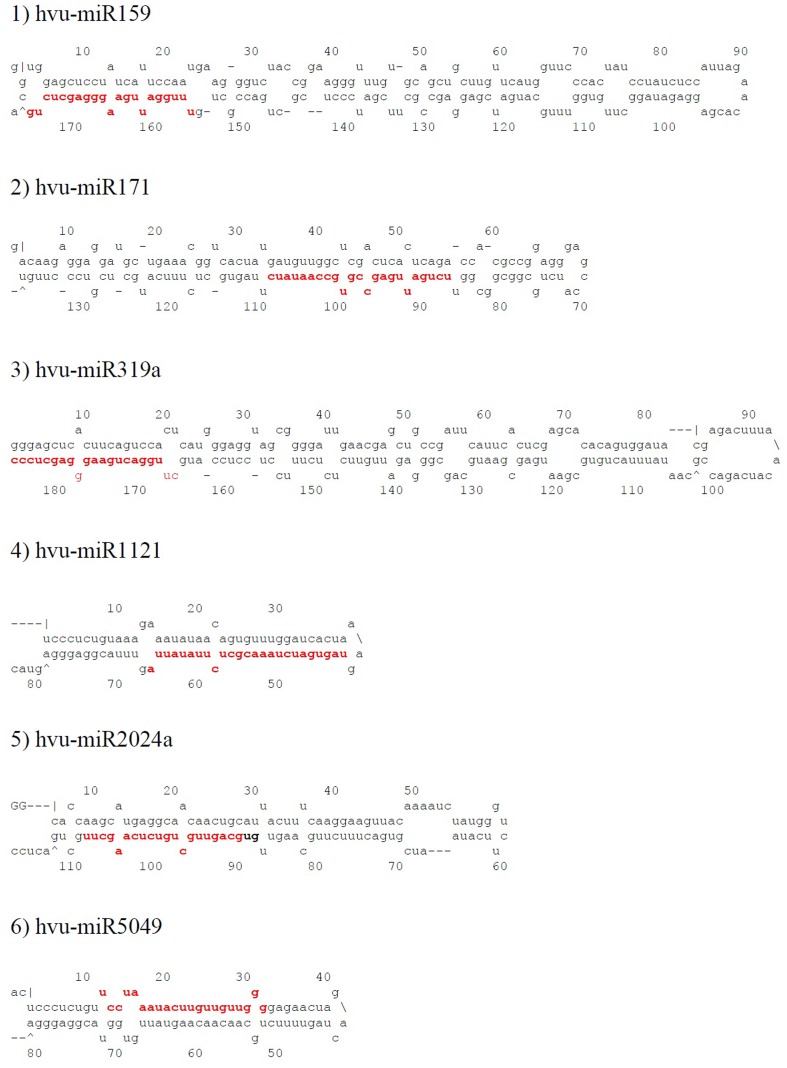
The secondary stem-loop structures of several identified miRNAs in barley. Mature miRNA sequences are marked in red color.

According to sequence similarity to known plant miRNAs, 31 known and 3 new miRNAs were identified. Previously, miR157, miR165, and miR319 have been identified in other plant species, but so far they have been undetermined in barley. Identified miRNAs in barley were located on either arm of the predicted pre-miRNA sequences. Of the 34 identified *H.vulgare* miRNAs, 47% of mature sequences were located in the 5′ arm of pre-miRNAs, while 53% were situated in the 3′ arm (Fig. 1; Fig. S1). The majority of these miRNAs were 21 nt long, followed by 22 nt, 20 nt and 23 nt, respectively (Table 1), which is consistent with miRNAs from other plant species [23], [36]. In addition, our study showed that the average of MFEI was 0.86, which is higher than that of other types of RNA molecules such as tRNAs (0.64), rRNAs (0.59) and mRNAs (0.62–0.66) (Table 1) [29], [37], [38], [39].

**Table 1:** Barley miRNAs and features identified by high-throughput sequencing.

miRNA name	Sequence (5′–3′)	LM	LP	MFEI	GC%	ΔG
hvu-mir-156	UGACAGAAGAGAGAGAGCAC	20	178	0.71	65.0	−83.20
hvu-mir-157	UUGACAGAAGAGAGUGAGCAC	21	85	1.12	55.0	−52.40
hvu-mir-159	UUUGGAUUGAAGGGAGCUCUG	21	178	0.93	52.0	−86.30
hvu-mir-160	UGCCUGGCUCCCUGUAUGCCA	21	98	0.95	60.0	−56.00
hvu-mir-164	UGGAGAAGCAGGGCACUUGCU	21	75	0.74	61.0	−34.10
hvu-mir-165	CCGCGACUGCCCCAUCCUCA	20	100	0.51	62.0	−31.90
hvu-mir-166	CCGGACCAGGCUUCAUUCCCA	21	61	0.34	59.0	−12.50
hvu-mir-168	GAUCCCGCCUUGCACCAAGUGAAU	24	106	0.81	75.0	−64.40
hvu-mir-169	AAGCCAAGGAUGAGUUGCCUG	21	83	0.80	45.0	−30.10
hvu-mir-171	UGAUUGAGCCGUGCCAAUAUC	21	137	0.97	55.0	−73.20
hvu-mir-172c	AGGAUCUUGAUGAUGCUGCUG	21	54	0.60	41	−13.40
hvu-mir-319a	UUGGACUGAAGGGAGCUCCC	20	186	0.90	52.0	−87.70
hvu-mir-319c	UUGGAAUGAAGGGAGCUCAA	20	78	0. 55	45.0	−19.60
hvu-mir-397	CCGUUGAGUGCAGCGUUGAUG	21	133	0. 98	67.0	−74.90
hvu-mir-399	UGCCAAAGGAGAUUUGCCCCG	21	113	0.65	46.0	−34.20
hvu-mir-408	CUGCACUGCCUCUUCCCUGGC	21	149	0.80	56.0	−67.50
hvu-mir-444b	UGCAGUUGCUGUCUCAAGCUU	21	121	1.01	45.0	−55.20
hvu-mir-1120	ACAUUCUUAUAUUAUGGGACGGAG	24	84	1.36	36.0	−41.30
hvu-mir-1121	AGUAGUGAUCUAAACGCUCUUA	22	83	1.53	36.0	−45.90
hvu-mir-1122	UUUGUACAUCCGUAUGUAGU	20	120	1.28	33.0	−50.70
hvu-mir-1126	UCCACUAUGGACUACAUACGGAG	23	120	1.28	33.0	−50.70
hvu-mir-2004	UUUGUUUUUAUGUUAUUUUGUGAA	24	78	0.74	29.0	−16.90
hvu-mir-2007	CAAGAUAUUGGGUAUUUUUGUC	22	54	1.59	30.0	−25.90
hvu-mir-2014	AGCAAACAUAUCUGAGCACA	22	109	0.60	49.0	−32.20
hvu-mir-2019	CGGGUCGGCGCUGCAUGCGGC	21	71	0.53	65.0	−24.70
hvu-mir-2023a	UUUUGCCGGUUGAACGACCUCA	22	113	0.74	55.0	−46.00
hvu-mir-2024a	GCAGUUGCUGUCUCAAGCUU	20	118	1.02	44.0	−53.40
hvu-mir-2906	AACGGGCCGCUGCACAACUGG	21	254	0.77	63.0	−123.9
hvu-mir-2911	UAGUUGGUGGAGCGAUUUGUC	21	71	0.56	49.0	−19.6
hvu-mir-2914	CAUGGUGGUGACGGGUGACGGAG	23	63	0.61	56.0	−21.8
hvu-mir-5048	UAUUUGCAGGUUUUAGGUCUAA	22	354	0.88	31.0	−96.90
hvu-mir-5049	UCCUAAAUACUUGUUGUUGGG	21	81	1.37	43.0	−47.80
hvu-mir-5051	UUUGGCACCUUGAAACUGGGA	21	105	1.20	49.0	−61.90
hvu-mir-5052	ACCGGCUGGACGGUAGGCAUA	21	175	0.89	54.0	−85.00
hvu-mir-5066	AAGUGUAUAUGUGGAGUGUCU	21	80	0.33	44.0	−11.70

LM: length of the mature miRNA; LP: length of the miRNA precursor sequence; MFEI: Minimal folding free energy index.

### Boron Stress Induced Aberrant Expression of miRNAs in Barley

To identify the response of barley miRNAs to B treatment, we compared the expression profile of miRNAs between treated and untreated groups. The reads were normalized on the basis of transcripts per million obtained from high-throughput sequencing (Table 2). Several conserved miRNAs (such as miR160 and miR171) and non-conserved miR5141 were found abundantly in both libraries, but many others were detected with only a few in both libraries or could not be found in either library. We also found that some miRNAs are only expressed in either root or leaf tissues. The miR156c and miR319a were highly expressed in root, whereas miR408 was only detected in leaf. In addition, some miRNAs such as miR156, miR169, miR172, and miR1121 were highly expressed in root but miR2004 was highly expressed in leaf. Expression of most miRNAs was significantly changed in a tissue-specific manner under boron stress whereas the remaining miRNAs were found to be responsive in both tissues. In root tissue responding to boron stress, miR165, miR2004, and miR5051 were up-regulated whereas miR444b and miR2024a were down-regulated. miR156, miR169c, miR171, miR171a, miR444a, miR444c, miR2023a were up-regulated while miR156d, miR397, miR408, miR1121, miR2014, miR5049, miR5141, miR5180, and miR5180a were down-regulated in leaf tissue upon boron stress. In addition, some miRNAs, such as miR172, miR399, miR2021, miR5053 and miR5066 were expressed in both root and leaf (Table 3).

**Table 2:** The normalized read counts of the pre-miRNAs in each sample.

miRNA name	50 µM Broot reads	1000 µM Broot reads	50 µM Bleaf reads	1000 µM Bleaf reads
hvu-miR156	146	232	28	81
hvu-miR156a/miR156b/miR156r	874	848	199	139
hvu-mir156c	22	21	0	0
hvu-mir157	46	25	87	43
hvu-miR159	746	886	196	339
hvu-miR160	211	236	4	4
hvu-miR160o	525	473	190	283
hvu-miR164a	120	177	28	20
hvu-miR165	104	221	58	78
hvu-miR166c	80	146	44	76
hvu-miR168	634	890	125	166
hvu-miR169	1711	937	192	263
hvu-miR169c	3	3	3	19
hvu-miR171	1450	1289	477	1090
hvu-miR171a	264	205	26	71
hvu-miR172	1473	699	149	651
hvu-miR319c	31	26	5	4
hvu-miR319/miR319a	171	211	0	0
hvu-miR397	58	51	15	2
hvu-miR399	124	35	31	94
hvu-miR408	0	0	130	8
hvu-miR444a	562	926	4	21
hvu-miR444b	83	26	14	21
hvu-miR444c	236	151	36	91
hvu-miR1120	869	1261	468	797
hvu-miR1121	2237	2115	31	11
hvu-miR1122	170	277	156	113
hvu-miR2004	4	9	118	77
hvu-miR2014	26	22	5	2
hvu-miR2021	9	22	18	6
hvu-miR2023a	38	70	34	69
hvu-miR2024a	83	26	14	21
hvu-miR2906	80	85	120	106
hvu-miR5048	1019	1169	217	218
hvu-miR5049	37	43	26	7
hvu-miR5051	35	73	27	35
hvu-miR5053	277	733	126	20
hvu-miR5064	248	250	116	159
hvu-miR5066	8	16	30	4
hvu-miR5141	993	1247	1199	175
hvu-miR5052	0	0	8	0
hvu-miR5180a/miR5180b	278	240	25	2

The mapped read counts of each pre-miRNAs were normalized in terms of the length of pre-miRNA and total read numbers according to RPKM method (Reads Per kb per Million reads) [61].

**Table 3:** The expression level of boron -responsive miRNAs**_Ψ_** from highly B treated and control B applied barley leaf and root tissues.

miRNA name	L−B expressed	L+B expressed	Fold change (Up/Down)	R−B expressed	R+B expressed	Fold change (Up/Down)
miR156	28	81	↑2-fold (up-regulated)	146	232	Not significantly changed
miR156d	87	43	↓2-fold (down-regulated)	46	25	Not significantly changed
miR165	58	78	Not significantly changed	104	221	↑2-fold (up-regulated)
miR169c	3	19	↑6-fold (up-regulated)	3	3	Not changed
miR171	477	1090	↑2-fold (up-regulated)	1450	1289	Not significantly changed
miR171a	26	71	↑2-fold (up-regulated)	264	205	Not significantly changed
miR172	149	651	↑4-fold (up-regulated)	1473	699	↓2-fold (down-regulated)
miR397	15	2	↓7-fold (down-regulated)	58	51	Not significantly changed
miR399	31	94	↑3-fold (up-regulated)	124	35	↓3-fold (down-regulated)
miR408	130	8	↓16-fold (down-regulated)	Not detected in root library		
miR444a	4	21	↑5-fold (up-regulated)	562	926	Not significantly changed
miR444b	14	21	Not significantly changed	83	26	↓3-fold (down-regulated)
miR444c	36	91	↑2-fold (up-regulated)	236	151	Not significantly changed
miR1121	31	11	↓2-fold (down-regulated)	2237	2115	Not significantly changed
miR2004	118	77	Not significantly changed	4	9	↑2-fold (up-regulated)
miR2014	5	2	↓2-fold (down-regulated)	26	22	Not significantly changed
miR2021	18	6	↓3-fold (down-regulated)	9	22	↑2-fold (up-regulated)
miR2023a	34	69	↑2-fold (up-regulated)	38	70	Not significantly changed
miR2024a	14	21	Not significantly changed	83	26	↑3-fold (up-regulated)
miR5049	26	7	↓3-fold (down-regulated)	37	43	Not significantly changed
miR5051	27	35	Not significantly changed	35	73	↑2-fold (up-regulated)
miR5053	126	20	↓6-fold (down-regulated)	277	733	↑2-fold (up-regulated)
miR5066	30	4	↓7-fold (down-regulated)	8	16	↑2-fold (up-regulated)
miR5141	1199	175	↓6-fold (down-regulated)	993	1247	Not significantly changed
miR5180a/miR5180b	25	2	↓12-fold (down-regulated)	278	240	Not significantly changed

L−B, B-free leaf; L+B, B-treated leaf; R−B, B-free root; R+B, B-treated root (**_Ψ_** miRNAs with fold change over 2).

### Target Identification of miRNAs in Barley Using Degradome Analysis

**Figure 2:**
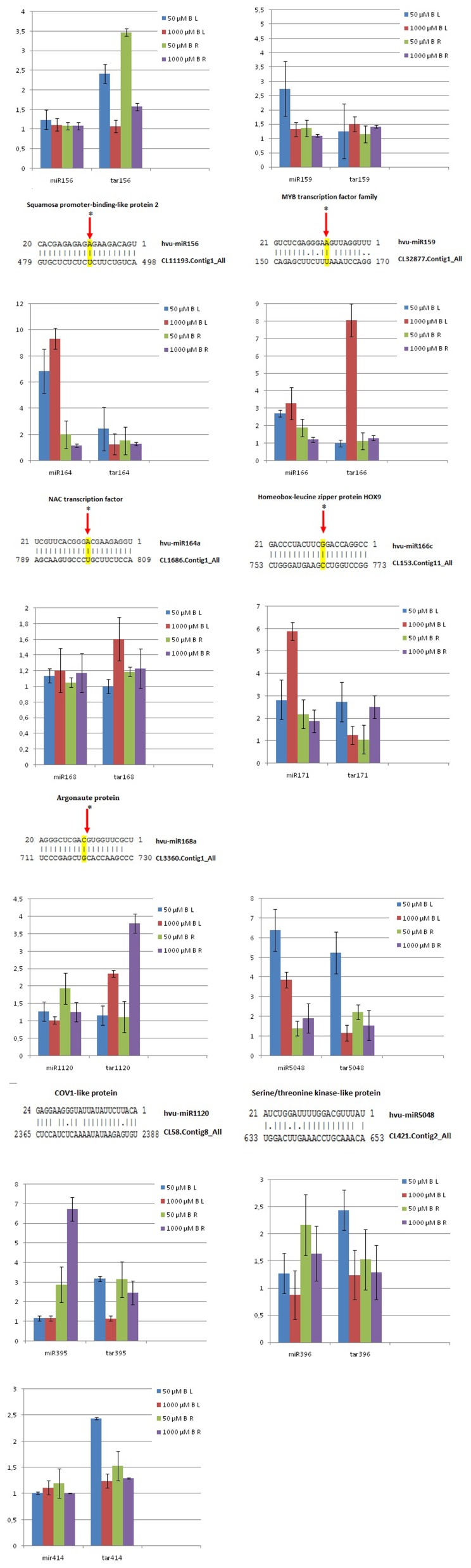
Expression levels of selected miRNAs and targets in leaf and root tissues in response to boron stress. Target plots of miRNA targets validated by degradome analysis (cleavage site are red letter) (B: Boron, L: Leaf, R: Root, miR: miRNA name, tar: miRNA target gene).

A total of 934 genes targeted by miRNAs were identified in barley by CleaveLand (v2.0) (Table 4). However, we could not identify the cleavage signature for some of the known miRNAs. The miRNA guided cleavage sites by degradome analysis are shown in Fig. 2 and Table S1. According to the results of blastn analysis of the identified miRNA targets, many of the targets were homologous to conserved target genes existing in other plants species; these targets included squamosa promoter-binding protein, auxin response factor (ARF), MYB transcription factor family, AP-2 Transcription Factors, NAC transcription factor (NAC), AGO1, and class III homeodomain-leucine zipper (HD-ZIP III) proteins. Most of these targets were found to be responsible for plant growth and response to environmental changes. For example, the target transcript of miR168 was ARGONAUTE1 protein (AGO1) family protein, which functions in plant development and in response to stress stimulus, such as NaCl and mannitol stress in rice. [40].

**Table 4:** Barley miRNA targets identified by degradome sequencing.

miRNA name	Target gene name	Target gene accesssion	Target gene number	Cleavage site
hvu-miR156/hvu-miR157	Squamosa promoter-binding-like protein (SLP)	CL11026.Contig1_All	12	789
		CL11193.Contig1_All	11	489
		CL13226.Contig1_All	3	613
		CL38155.Contig1_All	12	248
hvu-miR159/hvu-miR159a/hvu-miR159b	MYB transcription factor family	CL32877.Contig1_All	7	161
hvu-miR160	Auxin response factor (ARF)	CL7269.Contig1_All	13	232
hvu-miR164a/hvu-miR164b	NAC transcription factor (NAC)	CL1686.Contig1_All	15	800
		CL3897.Contig1_All	15	801
		CL6305.Contig2_All	13	967
		CL8731.Contig1_All	13	1013
		CL19527.Contig1_All	10	311
		Unigene5170_All	15	868
		Unigene29351_All	15	953
hvu-miR165/hvu-miR166c	Class III Homeodomain-leucine zipper (HD-ZIPIII) proteins	CL153.Contig8	16	452
		CL153.Contig11	17	764
hvu-miR168a(3p)/hvu-miR168b(3p)	Argonaute protein (AGO1)	CL3360.Contig1	16	720
hvu-miR169	Nuclear transcription factor Y subunit (NF-Y)	CL5590.Contig1	17	943
		CL3849.Contig1	15	1123
		CL2801.Contig1	13	913
hvu-miR172c/hvu-miR172d	AP-2 Transcription Factors	CL27047.Contig1	10	906
		Unigene3420	10	936
hvu-miR319a/hvu-miR319c	MYB transcription factor family	CL32877.Contig1	7	201
		CL2226.Contig1	9	527
hvu-miR397	Laccase mRNA	CL1278.Contig5	14	547
hvu-miR399	Phosphate transporter 2 (PHO2) and Putativeubiquitin conjugating enzyme (UBC)	CL876.Contig1	18	1629
		CL876.Contig4	18	813
hvu-miR408	Heterotrimeric G protein alpha subunit orATPase family gene 1 (AFG1)	CL30341.Contig1_All	14	undetermined
		Unigene31703_All	11	undetermined
hvu-miR444/hvu-miR444a/hvu-miR444b/hvu-miR444c	MADS-box transcription factor	CL1260.Contig1	19	633
		CL3271.Contig2	20	344
hvu-miR1120	COV1-like protein	CL58.Contig8_All	16	undetermined
hvu-miR1121	Serine/threonine protein kinase	CL3697.Contig1_All	13	undetermined
		Unigene28145_All	14	undetermined
hvu-miR1122	Phospholipase A2 and Universal stress protein (USP) and WIR1	CL1.Contig23_All	14	undetermined
		CL2147.Contig2_All	13	undetermined
		CL2301.Contig1_All	3	undetermined
hvu-miR1126	Zinc finger ccch domain-containing protein	CL6067.Contig1_All	12	undetermined
		CL6067.Contig2_All	10	undetermined
		CL6067.Contig3_All	10	undetermined
hvu-miR2004	PHD finger family protein,AP-1 complex subunit,Subtilase family protein,Tetratricopeptide repeat-containing protein andTranscription elongation factor (TFIIS) family protein	CL1242.Contig3_All	18	undetermined
		CL6239.Contig1_All	11	undetermined
		CL904.Contig1_All	14	undetermined
		CL162.Contig5_All	8	undetermined
		CL17869.Contig1_All	11	undetermined
hvu-miR2007	Protein phosphatase and Serine/arginine repetitive matrix protein	CL2929.Contig1_All	12	undetermined
		CL6012.Contig1_All	11	undetermined
hvu-miR2014	Phospholipid-translocating ATPase, GTP-binding protein, Ethylene responsive factor and Transcription factor jumonji	CL283.Contig1_All	12	undetermined
		CL7041.Contig1_All	17	undetermined
		CL2423.Contig1_All	8	undetermined
		CL3225.Contig1_All	13	undetermined
hvu-miR2019	Tubulin-tyrosine ligase family	CL326.Contig1_All	14	undetermined
hvu-miR2021	Rough sheath 2-interacting KH domain protein (RIK), Lysophosphatidylcholine Acyltransferase, Respiratory burst oxidase-like protein F2 and Cytochrome P450	CL527.Contig3_All	5	undetermined
		CL318.Contig4_All	15	undetermined
		CL2680.Contig1_All	12	undetermined
		Unigene27511_All	8	undetermined
hvu-miR2024a	MADS box protein-like protein and Zinc finger family protein	CL3271.Contig2_All	20	undetermined
		CL9100.Contig1_All	12	undetermined
hvu-miR2906	(E)-beta-caryophyllene/beta-elemene synthase	CL40097.Contig1_All	7	undetermined
		Unigene30593_All	7	undetermined
hvu-miR2910	glycine rich protein 3, glyceraldehyde-3-phosphate dehydrogenase, cytosoli, phosphatidylinositol-4-phosphate 5-kinase 9 and ubiquitin-associated protein	CL40314.Contig1_All	15	undetermined
		CL386.Contig2_All	10	undetermined
		CL5067.Contig2_All	12	undetermined
		Unigene11586_All	16	undetermined
hvu-miR2914	Senescence-associated protein, CBL-interacting protein kinase 21	CL8337.Contig1_All	11	undetermined
		CL660.Contig7_All	11	undetermined
hvu-miR2916	Senescence-associated protein	CL8337.Contig1_All	10	undetermined
hvu-miR5048	RPG1, Serine/threonine protein kinase, NAC domain-containing protein 18 and Serine/threonine kinase-like protein	CL26250.Contig1_All	12	undetermined
		CL2067.Contig1_All	16	undetermined
		CL5978.Contig2_All	14	undetermined
		CL421.Contig2_All	14	undetermined
hvu-miR5049	Tubby protein-like	CL9685.Contig1_All	9	undetermined
hvu-miR5052	Cyclophilin	CL27515.Contig1_All	9	undetermined
hvu-miR5053	Chlorophyll a/b-binding protein and Predicted protein	CL40448.Contig1_All	13	undetermined
		CL33769.Contig1_All	1	undetermined
hvu-miR5056	RNA polymerase beta subunit	CL179.Contig1_All	7	undetermined
hvu-miR5066	Carbohydrate transporter/sugar porter/transporter and Serine/threonine protein kinase	CL21592.Contig1_All	3	undetermined
		CL6.Contig12_All	13	undetermined

### qRT-PCR Validation and Measurement of *H. vulgare* miRNA Levels and their Targets

Eleven identified barley miRNAs and their targets were further investigated using qRT-PCR. Both conserved barley miRNAs (miR156, miR159, miR164, miR166, miR168, miR171, miR395 and miR396) and non-conserved barley miRNAs (miR1120 and miR5048) were detected. The expression levels of barley miRNAs and their targets were comparatively shown in Fig. 2. The miR159, miR164, miR166, miR171, and miR414 were induced in leaf, but were inhibited in root tissues exposed to boron stress. Although miR168 was induced, miR159, miR396, miR1120 and miR5048 were inhibited in both root and leaf upon excess boron exposure. The targets of miR159 and miR1120 were found to be up-regulated in both root and leaf upon boron stress, but miR395 and miR5048 target genes were down-regulated in root but remained at the same levels in leaf tissue upon boron stress. Additionally, miR171 target gene was down-regulated in leaf but up-regulated in root upon boron stress (Fig. 2).

### Gene Ontology (GO) Analysis

According to the gene ontology analysis, the predicted targets were classified into three main categories: biological processes, cellular components, and molecular functions (Table 5). Of these, cellular and metabolic process in biological process, cell and organell part in cellular component, and binding and catalytic activity in molecular function were the most established categories.

**Table 5:** Gene Ontology analyses indicate that miRNAs and target in related to biological process, cellular component, molecular function process.

miRNAs	GO Biological Process	GO Cellular Component	GO Molecular Function	Target Gene	Target Description
hvu-miR156hvu-miR157	–	Organelle (plastid) and cellular part (nucleus)	DNA binding	CL13226.Contig1_AllCL11026.Contig1_AllCL11193.Contig1_AllCL38155.Contig1_All	Squamosa promoter-binding protein
hvu-miR159hvu-miR159ahvu-miR159b	–	Intracellular organelleNucleus	Nucleic acid (DNA) binding	CL32877.Contig1_All	MYB family transcription factor (GAMyb transcription factor family)
hvu-miR160	Response to stimulusCellular processBiological regulationSignalingMetabolic process	Organelle and nucleus	Nucleic acid (DNA) binding	CL7269.Contig1_All	Auxin response factor (ARF)
hvu-miR164ahvu-miR164b	–	–	Nucleic acid (DNA) binding	CL6305.Contig2_AllCL1686.Contig1_AllUnigene29351_AllCL19527.Contig1_AllCL3897.Contig1_AllCL8731.Contig1_AllUnigene5170_All	NAC transcription factor (NAC)
hvu-miR165hvu-miR166c	Biological regulationCellular processMetabolic process	Intracellular organelleNucleus	Nucleic acid bindingTranscription factor activity	CL153.Contig8_AllCL153.Contig11_All	Class III Homeodomain-leucine zipper (HD-ZIP III) proteins
hvu-miR168a (3p)hvu-miR168b (3p)	Multicellular organismal processReproductionBiological regulationImmune system processResponse to stimulusMetabolic processCellular processDevelopmental process	NucleusCytosol	Nucleic acid bindingCatalytic activity	CL3360.Contig1_All	AGO1 (ARGONAUTE 1)
hvu-miR169	Biological regulationMetabolic processCellular process	Macromolecular complexMembrane-enclosed lumenMembrane-bounded organelleNucleoplasm part	Nucleic acid binding	CL5590.Contig1_AllCL3849.Contig1_AllCL2801.Contig1_All	Nuclear transcription factor Y subunit (NF-Y)
hvu-miR172chvu-miR172d	Biological regulationMetabolic processCellular process	Intracellular organelleNucleus	Nucleic acid bindingCatalytic activity	CL27047.Contig1_AllUnigene3420_All	AP-2 Transcription Factors
hvu-miR319ahvu-miR319c	–	NucleusIntracellular membrane-bounded organelle	Nucleic acid binding	CL32877.Contig1_AllCL2226.Contig1_All	MYB transcription factor family
hvu-miR397	Metabolic processCellular process	Extracellular regionOrganelleCytoplasmic vesicle	Nucleic acid bindingCatalytic activity	CL1278.Contig5_All	Laccase mRNA
hvu-miR399	Biological regulationCellular processLocalizationMetabolic processResponse to stimulus	–	Catalytic activity	CL876.Contig1_AllCL876.Contig4_All	Phosphate transporter 2 (PHO2) orPutative ubiquitin conjugating enzyme (UBC)
hvu-miR408	Response to pheromone	Organelle (mitochondrion)Cytoplasmic part	BindingCatalytic activity	CL30341.Contig1_AllUnigene31703_All	Heterotrimeric G protein alpha subunit or ATPase family gene 1 (AFG1)
hvu-miR444hvu-miR444ahvu-miR444bhvu-miR444c	Biological regulationCellular processMetabolic process	Organelle and nucleus	BindingCatalytic activity	CL1260.Contig1_AllCL3271.Contig2_All	MADS-box transcription factor
hvu-miR1120	–	–	–	CL58.Contig8_All	COV1-like protein
hvu-miR1121	Cellular processMetabolic process		BindingCatalytic activity	CL3697.Contig1_AllUnigene28145_All	Serine/threonine protein kinase
hvu-miR1122	Response to stimulus	Organelle (mitochondrion)Membrane	–	CL1.Contig23_AllCL2147.Contig2_AllCL2301.Contig1_All	Phospholipase A2 or Universal stress protein (USP) or WIR1
hvu-miR1126		Cellular componentMembrane	BindingCatalytic activity	CL6067.Contig1_AllCL6067.Contig2_AllCL6067.Contig3_All	Zinc finger ccch domain-containing protein
hvu-miR2004	Biological regulationCellular processLocalizationMetabolic process	CellExtracellular regionMacromolecular complexOrganelle	BindingCatalytic activityTranscription regulatory activity	CL1242.Contig3_AllCL6239.Contig1_AllCL904.Contig1_AllCL162.Contig5_AllCL17869.Contig1_All	PHD finger family protein or AP-1 complex subunit or Subtilase family protein or Tetratricopeptide repeat-containing protein or Transcription elongation factor (TFIIS) family protein
hvu-miR2007	Cellular processMetabolic process	CellOrganelle (Plastid)	BindingCatalytic activity	CL2929.Contig1_AllCL6012.Contig1_All	Protein phosphatase or Serine/arginine repetitive matrix protein
hvu-miR2014	Biological regulationCellular processLocalizationMetabolic processResponse to stimulusSignaling	Organelle (mitochondrion)Cytoplasmic part	BindingCatalytic activityMolecular transducer activityTransporter activity	CL283.Contig1_AllCL7041.Contig1_AllCL2423.Contig1_AllCL3225.Contig1_All	Phospholipid-translocating ATPase or GTP-binding protein or Ethylene responsive factor or Transcription factor jumonji
hvu-miR2019	Cellular processMetabolic process	CellOrganelle (Chlroplast)	Catalytic activity	CL326.Contig1_All	Tubulin-tyrosine ligase family
hvu-miR2021	Cellular processMetabolic process	Cell partNucleusIntracellular membrane-bounded organelle	Antioxidant activityBindingCatalytic activityElectron carrier activity	CL527.Contig3_AllUnigene27511_AllCL318.Contig4_AllCL2680.Contig1_All	Rough sheath 2-interacting KH domain protein (RIK) or Lysophosphatidylcholine Acyltransferase or Respiratory burst oxidase-like protein F2 or Cytochrome P450
hvu-miR2024a	Biological regulationCellular processMetabolic process	Intracellular organelleMembrane-bounded organelleNucleus	Binding	CL3271.Contig2_AllCL9100.Contig1_All	MADS box protein-like protein or Zinc finger family protein
hvu-miR2906	–	–	–	CL40097.Contig1_AllUnigene30593_All	(E)-beta-caryophyllene/beta-elemene synthase
hvu-miR2910	Biological regulationCellular processDevelopmental processMetabolic processMulticellular organismal processReproduction	Cellular componentCytoplasmIntracellular part	BindingCatalytic activity	CL40314.Contig1_AllCL386.Contig2_AllUnigene11586_AllCL5067.Contig2_All	Glycine rich protein 3 or Glyceraldehyde-3-phosphate dehydrogenase, cytosoli or Phosphatidylinositol-4-phosphate 5-kinase 9 or Ubiquitin-associated protein
hvu-miR2911	–	Intracellular organelleMembrane-bounded organelleMitochondrion	Binding	CL17424.Contig1_AllCL23524.Contig1_All	ASF/SF2-like pre-mRNA splicing factor SRP32 or Hydroxyproline-rich glycoprotein family protein
hvu-miR2914hvu-miR2916	Biological regulationCellular processMetabolic processResponse to stimulusSignaling	Cytoplasmic partIntracellular membrane-bounded organellePlastid	BindingCatalytic activity	CL8337.Contig1_AllCL660.Contig7_All	Senescence-associated protein or CBL-interacting protein kinase 21
hvu-miR5048	Cellular processMetabolic process	–	BindingCatalytic activity	CL26250.Contig1_AllCL2067.Contig1_AllCL5978.Contig2_AllCL421.Contig2_All	RPG1 or Serine/threonine protein kinase or NAC domain-containing protein 18 or Serine/threonine kinase-like protein
hvu-miR5049	Biological regulationCellular processMetabolic process	–	Sequence-specific DNA binding transcription factor activity	CL9685.Contig1_All	Tubby protein-like
hvu-miR5052	Cellular processMetabolic process	–	BindingCatalytic activityElectron carrier activity	CL27515.Contig1_All	Cyclophilin
hvu-miR5053	Cellular processMetabolic process	ChloroplastMembrane	–	CL40448.Contig1_All	Chlorophyll a/b-binding protein or Predicted protein
hvu-miR5056	–	–	–	CL179.Contig1_All	RNA polymerase beta subunit
hvu-miR5066	Cellular processMetabolic process	Cell partMembrane	BindingCatalytic activity	CL21592.Contig1_AllCL6.Contig12_All	Carbohydrate transporter/sugar porter/transporter or Serine/threonine protein kinase

## Discussion

High-throughput sequencing technology has currently been successfully applied to identify miRNAs at whole genome scale in several plant species, including: soybean [41], [42], peanut [43], [44], barley [21], [45], poplar [46], olive [47], *Medicago* [48], grapevine [49], rice [50], and cucumber [23]. However, almost all of the previous studies have been performed under normal growth conditions, few are associated with stress conditions. Li et al. [41] reported soybean miRNAs under three stress treatments (drought, salinity, and alkalinity) via high-throughput sequencing. Drought stress responsive miRNAs shows differential expression in response to heat stress in *Populus euphratica* and wheat [46], [51]. In this study, we constructed RNA libraries from barley leaves and roots treated with boron stress compared to control conditions to idnetify boron stress-responsive miRNAs in barley using high-throughput sequencing.

Boron treatment affected the expression profiles of miRNAs in barley leaf and root tissues. The most striking oneswith 16-fold and 12-fold changes were miR408 and miR5180, respectively. The remaining changes in the expression of miRNAs ranged between 2- to 7-fold (Table 3).

Recently, miR408 was identified in barley, which targets Cu-binding domain containing chemocyanin and blue copper protein [19]. In this study, we found that miR408 also potenially targets heterotrimeric G protein alpha (α) subunit and ATPase family gene 1 (AFG1). Heterotrimeric G proteins and ATPase gene family plays significant roles in signal transduction pathways in plants [52], [53], [54]. Fujisawa et al. [55] reported that suppression of α subunit gene expression causes abnormal morphology in rice. In response to water deficit, miR398 and miR408 were induced in *Medicago truncatula* [56]. In addition, expression of miR408 upon drought stress in barley was found to be induced in leaves, but unchanged in roots [19]. However, in *Oryza sativa*, miR408 expression was reported as 2.76-fold down-regulated 12 days after water withholding at tillering stage upon drought stress using microarray analysis [40]. In our study, expression of miR408 was down-regulated significantly (16-fold) upon excess boron treatment in barley leaves.

Previous studies reported the miRNA expression in a species-specific or tissue-specific manner [57], [58]. miR168, miR319, miR396, and miR397 were induced by drought in *Arabidopsis thaliana* but were suppressed in *Oryza sativa* [59]. Additionally, the expression of miR399 was induced in shoots upon phosphate deficiency treatment, but it was accumulated in both shoots and roots [57]. In barley, miR166 was up-regulated in leaves, but was down-regulated in roots; miR171 level was induced in leaves, but it was not affected in roots [19]. In our study, miR169c, miR171, and miR399 were up-regulated in leaves whereas miR397, miR444b were down-regulated in roots after exposure to high B concentration. The miR172 was down-regulated 2-fold in roots but up-regulated 4-fold in leaves in response to boron stress. The miR169c and miR171 was determined to be 6-fold up-regulated and 2-fold up-regulated in leaves under boron stress, respectively. In *Medicago truncatula*, miR169 and miR172 were up-regulated but miR171 and miR390 were down-regulated upon mercury exposure [16]. Similarly, miR171 was down-regulated but miR172 was up-regulated by cadmium exposure in *Brassica napus* [15]. However, in response to Al^3+^ treatment, miR171 was up-regulated in *Medicago truncatula* [14].

Our study demonstrated that boron stress inhibited miR156a expression in barley leaves. However, we did not detect its expression in roots. In addition, the target of miR156a, SBP protein gene, was down-regulated in stressed leaves, but was unaltered in roots in response to boron stress (Fig. 2). This result is similar to the prvious report [19], whereas not affected in roots upon drought stress. Expression of miR156 has been investigated in many studies as down-regulated in *Oryza sativa*, *Zea mays*, *Populus tremula*, *Populus trichocarpa* in response to drought stress, salt stress, cold stress, mechanical stress, while up-regulated in *Arabidopsis thaliana*, *Triticum aestivum*, *Nicotiana tabacum* upon salt stress, heat stress, viral infection, respectively [59]. Our study indicates that miR156 was also boron stress responsive in leaves upon excess boron treatment.

For better understanding of the functions of miRNAs, gene ontology analysis for miRNA target transcripts was performed. Sixty genes targted by 34 miRNAs were found to be involved in 77 biological processes. These major processes are as follows: biological regulation, metabolic process, response to stimulus, cellular process, signaling, multicellular organismal process, reproduction, immune system process, developmental process, and localization. The most (24 out of 34) miRNAs participated in the cellular and metabolic processes, and the rest 12 miRNA families may be involved in other processes. For example, miR168 and miR2910 may have a role in plant reproduction, whereas miR160, miR2014 and miR2916 might be associated with signal transduction. Using gene ontology analysis, Mao et al. [23] reported that abscisic acid and salicylic acid stimulus might be regulated by miR159 and miR858 in cucumber. Furthermore, according to gene ontology analysis, 3 miRNAs (miR399, miR1122 and miR2014) were determined to be regulated in response to boron stress.

In conclusion, we identifed 32 known and 4 new barley miRNAs, as well as 934 target genes using recently developed degradome analysis. The majority of the identified miRNAs were significantly responsive to boron stress in barley. In particular, the signal transduction mechanism in leaves regulated by miR408 plays an important role in boron tolerance in barley consistent with previous reports [40], [60].

## Supporting Information

Figure S1 The sequences, additional properties, and stem-loop secondary structure of pre-microRNAs of *Hordeum vulgare*
(DOC)

Table S1 MicroRNA guided cleavage sites by degradome analysis.
(DOCX)

Table S2 Primers used for miRNA validation and measurement detected in this study.
(DOCX)

Table S3 Primers used for target mRNA validation and measurement detected in this study.
(DOCX)

## References

[1] Llave C, Kasschau KD, Rector MA, Carrington JC (2002) Endogenous and silencing associated small RNAs in plants. Plant Cell 14: 1605–1619. 12119378 10.1105/tpc.003210 PMC150710

[2] Carrington J, Ambros V (2003) Role of microRNAs in plant and animal development. Science 301: 336–338. 12869753 10.1126/science.1085242

[3] Bartel D (2004) MicroRNAs: genomics, biogenesis, mechanism, and function. Cell 116: 281–297. 14744438 10.1016/s0092-8674(04)00045-5

[4] Jones-Rhoades MW, Bartel DP, Bartel B (2006) MicroRNAs and their regulatory roles in plants. Annu Rev Plant Biol 57: 19–53. 16669754 10.1146/annurev.arplant.57.032905.105218

[5] Zhang BH, Wang QL, Pan XP (2007) MicroRNAs and their regulatory roles in animals and plants. J Cell Physiol 210(2): 279–289. 17096367 10.1002/jcp.20869

[6] Sunkar R, Chinnusamy V, Zhu JH, Zhu JK (2007) Small RNAs as big players in plant abiotic stress responses and nutrient deprivation. Trends Plant Sci 12: 301–309. 17573231 10.1016/j.tplants.2007.05.001

[7] Shukla LI, Chinnusamy V, Sunkar R (2008) The role of microRNAs and other endogenous small RNAs in plant stress responses. Biochim Biophys Acta-Gene Regul Mech 1779: 743–748. 10.1016/j.bbagrm.2008.04.004 18457682

[8] Eldem V, Okay S, Unver T (2013) Plant microRNAs: new players in functional genomics. Turk J Agric For 37: 1–21.

[9] Miwa K, Fujiwara T (2010) Boron transport in plants: co-ordinated regulation of transporters. Ann Bot 105: 1103–1108. 20228086 10.1093/aob/mcq044 PMC2887066

[10] Nable RO, Banuelos GS, Paull JG (1997) Boron toxicity. Plant and Soil 193: 181–198.

[11] Stangoulis JCR, Reid RJ (2002) Boron toxicity in plants and animals. In: Goldbach HE et al (eds) Boron in plant and animal nutrition. Kluwer Academic Press, New York 227–240.

[12] Reid RJ, Hayes JE, Post A, Stagoulis JCR, Graham RD (2004) A critical analysis of the causes of boron toxicity in plants. Plant Cell Environ 25: 1405–1414.

[13] Wang BL, Shi L, Li YX, Zhang WH (2010) Boron toxicity is alleviated by hydrogen sulfide in cucumber (*Cucumis sativus* L.) seedlings. Planta 231: 1301–1309. 20224946 10.1007/s00425-010-1134-9

[14] Zhou ZS, Huang SQ, Yang ZM (2008) Bioinformatic identification and expression analysis of new microRNAs from *Medicago truncatula* . Biochem Biophys Res Commun 374: 538–542. 18662674 10.1016/j.bbrc.2008.07.083

[15] Zhou ZS, Song JB, Yang ZM (2012) Genome-wide identification of *Brassica napus* microRNAs and their targets in response to cadmium. J Exp Bot 63(12): 4597–4613. 22760473 10.1093/jxb/ers136 PMC3421990

[16] Mendoza-Soto AB, Sánchez F, Hernández G (2012) MicroRNAs as regulators in plant metal toxicity response. Front Plant Sci 3: 105. 22661980 10.3389/fpls.2012.00105 PMC3356851

[17] Schulte D, Timothy JC, Andreas G, Langridge P, Matsumoto T, et al (2009) The international barley sequencing consortium–At the threshold of efficient access to the barley genome. Plant Physiol 149: 142–147. 19126706 10.1104/pp.108.128967 PMC2613708

[18] Sreenivasulu N, Graner A, Wobus U (2008) Barley genomics: An overview. Int J Plant Genomics 2008, 486258. 10.1155/2008/486258 PMC2276607 18382615

[19] Kantar M, Unver T, Budak H (2010) Regulation of barley miRNAs upon dehydration stress correlated with target gene expression. Funct Integr Genomics 10: 493–507. 20676715 10.1007/s10142-010-0181-4

[20] Dryanova A, Zakharov A, Gulick PJ (2008) Data mining for miRNAs and their targets in the *Triticeae* . Genome 51: 433–443. 18521122 10.1139/G08-025

[21] Schreiber AW, Jun Shi B, Yuan Huang C, Langridge P, Baumann U (2011) Discovery of barley miRNAs through deep sequencing of short reads. BMC Genomics 12: 129. 21352554 10.1186/1471-2164-12-129 PMC3060140

[22] Lv S, Nie X, Wang L, Du X, Biradar SS, et al (2012) Identification and Characterization of MicroRNAs from Barley (*Hordeum vulgare* L.) by High-Throughput Sequencing. Int J Mol Sci 13: 2973–2984. 22489137 10.3390/ijms13032973 PMC3317698

[23] Mao W, Li Z, Xia X, Li Y, Yu J (2012) A Combined Approach of High-Throughput Sequencing and Degradome Analysis Reveals Tissue Specific Expression of MicroRNAs and Their Targets in Cucumber. PLoS ONE 7(3): e33040. 22479356 10.1371/journal.pone.0033040 PMC3316546

[24] Grabherr MG, Haas BJ, Yassour M, Levin JZ, Thompson DA, et al (2011) Full-length transcriptome assembly from RNA-Seq data without a reference genome. Nat Biotechnol 29: 644–652. 21572440 10.1038/nbt.1883 PMC3571712

[25] Zuker M (2003) Mfold web server for nucleic acid folding and hybridization prediction. Nucleic Acids Res 31: 3406–3415. 12824337 10.1093/nar/gkg595 PMC169194

[26] Zhang BH, Pan XP, Cox SB, Cobb GP, Anderson TA (2006) Evidence that miRNAs are different from other RNAs. Cell Mol Life Sci 63(2): 246–254. 16395542 10.1007/s00018-005-5467-7 PMC11136112

[27] Zhang B, Pan XP, Cobb GP, Anderson TA (2006) Plant microRNA: a small regulatory molecule with big impact. Dev Biol 289: 3–16. 16325172 10.1016/j.ydbio.2005.10.036

[28] Zhang B, Wang QL, Wang K, Pan X, Liu F, et al (2007) Identification of cotton microRNAs and their targets. Gene 397: 26–37. 17574351 10.1016/j.gene.2007.03.020

[29] Yin Z, Li C, Han X, Shen F (2008) Identification of conserved microRNAs and their target genes in tomato (*Lycopersicon esculentum*). Gene 414: 60–66. 18387754 10.1016/j.gene.2008.02.007

[30] Unver T, Budak H (2009) Conserved microRNAs and their targets in model grass species *Brachypodium distachyon* . Planta 230: 659–669. 19585143 10.1007/s00425-009-0974-7

[31] Zhang Y (2005) miRU: an automated plant miRNA target prediction server. Nucleic Acids Res 33 (Web Server issue), W701–4. 10.1093/nar/gki383 PMC1160144 15980567

[32] Götz S, García-Gómez JM, Terol J, Williams TD, Nagaraj SH, et al (2008) High-throughput functional annotation and data mining with the blast2go suite. Nucleic Acids Res 36: 3420–3435. 18445632 10.1093/nar/gkn176 PMC2425479

[33] Varkonyi-Gasic E, Wu R, Wood M, Walton EF, Hellens RP (2007) Protocol: a highly sensitive RT-PCR method for detection and quantifi cation of microRNAs. Plant Method 3: 12. 10.1186/1746-4811-3-12 PMC2225395 17931426

[34] Unver T, Covert DN, Budak H (2009) Review of current methodological approaches for characterizing microRNAs in plants. Int J Plant Genomics 262463. 10.1155/2009/262463 PMC2760397 19834623

[35] Unver T, Bozkurt O, Akkaya MS (2008) Identification of differentially expressed transcripts from leaves of the boron tolerant plant *Gypsophila perfoliata* L. Plant Cell Rep. 27(8): 1411–1422. 10.1007/s00299-008-0560-7 18504585

[36] Unver T, Parmaksız I, Dündar E (2010) Identification of conserved micro-RNAs and their target transcripts in opium poppy (*Papaver somniferum* L.). Plant Cell Rep 29: 757–769. 20443006 10.1007/s00299-010-0862-4

[37] Unver T, Bakar M, Shearman RC, Budak H (2010) Genome-wide profiling and analysis of Festuca arundinacea miRNAs and transcriptomes in response to foliar glyphosate application. Mol Genet Genomics 283: 397–413. 20213187 10.1007/s00438-010-0526-7

[38] Zhang B, Pan X, Cannon CH, Cobb GP, Anderson TA (2006) Conservation and divergence of plant microRNA genes. Plant J 46(2): 243–259. 16623887 10.1111/j.1365-313X.2006.02697.x

[39] Zhang B, Pan X, Stellwag EJ (2008) Identification of soybean microRNAs and their targets. Planta 229(1): 161–182. 18815805 10.1007/s00425-008-0818-x

[40] Zhou L, Liu Y, Liu Z, Kong D, Duan M, et al (2010) Genome-wide dentification and analysis of drought-responsive microRNAs in *Oryza sativa* . J Exp Bot 61: 4157–4168. 20729483 10.1093/jxb/erq237

[41] Li H, Dong Y, Yin H, Wang N, Yang J, et al (2011) Characterization of the stress associated microRNAs in *Glycine max* by deep sequencing. BMC Plant Biol 11: 170. 22112171 10.1186/1471-2229-11-170 PMC3267681

[42] Song QX, Liu YF, Hu XY, Zhang WK, Ma B, et al (2011) Identification of miRNAs and their target genes in developing soybean seeds by deep sequencing. BMC Plant Biol 11: 5. 21219599 10.1186/1471-2229-11-5 PMC3023735

[43] Zhao CZ, Xia H, Frazier TP, Yao YY, Bi YP, et al (2010) Deep sequencing identifies novel and conserved microRNAs in peanuts (*Arachis hypogaea* L.). BMC Plant Biol 10: 3. 20047695 10.1186/1471-2229-10-3 PMC2826338

[44] Chi X, Yang Q, Chen X, Wang J, Pan L, et al (2011) Identification and Characterization of microRNAs from Peanut (*Arachis hypogaea* L.) by High-Throughput Sequencing. PLoS ONE 6: 11. 10.1371/journal.pone.0027530 PMC3217988 22110666

[45] Colaiacovo M, Subacchi A, Bagnaresi P, Lamontanara A, Cattivelli L, et al (2010) A computational-based update on microRNAs and their targets in barley (*Hordeum vulgare* L.). BMC Genomics 11: 595. 20969764 10.1186/1471-2164-11-595 PMC3091740

[46] Li B, Qin Y, Duan H, Yin W, Xia X (2011) Genome-wide characterization of new and drought stres responsive microRNAs in *Populus euphratica* . J Exp Bot 62: 3765–3779. 21511902 10.1093/jxb/err051 PMC3134338

[47] Donaire L, Pedrola L, de la Rosa R, Llave C (2011) High-Throughput Sequencing of RNA Silencing-Associated Small RNAs in Olive (*Olea europaea* L.). PLoS ONE 6: 11. 10.1371/journal.pone.0027916 PMC3225373 22140484

[48] Wang T, Chen L, Zhao M, Qiuying Tian Q, Hao Zhang W (2011) Identification of drought-responsive microRNAs in *Medicago truncatula* by genome-wide high-throughput sequencing. BMC Genomics 12: 367. 21762498 10.1186/1471-2164-12-367 PMC3160423

[49] Pantaleo V, Szittya G, Moxon S, Miozzi L, Moulton V, et al (2010) Identification of grapevine microRNAs and their targets using high-throughput sequencing and degradome analysis. Plant J 62: 960–976. 20230504 10.1111/j.0960-7412.2010.04208.x

[50] Li YF, Zheng Y, Addo Quaye C, Zhang L, Sain A, et al (2010) Transcriptome-wide identification of microRNA targets in rice. Plant J 62: 742–759. 20202174 10.1111/j.1365-313X.2010.04187.x

[51] Xin M, Wang Y, Yao Y, Xie C, Peng H, et al (2010) Diverse set of microRNAs are responsive to powdery mildew infection and heat stress in wheat (*Triticum aestivum* L.). BMC Plant Biol 10: 123. 20573268 10.1186/1471-2229-10-123 PMC3095282

[52] Ando S, Takumi S, Ueda Y, Ueda T, Mori N, et al (2000) *Nicotiana tabacum* cDNAs encoding alpha and beta subunits of a heterotrimeric GTP-binding protein isolated from hairy root tissues. Genes Genet Syst 75: 211–221. 11126570 10.1266/ggs.75.211

[53] Bussemer J, Chigri FC, Vothknecht U (2009) *Arabidopsis* ATPase family gene 1-like protein 1 is a calmodulin-binding AAA+-ATPase with a dual localization in chloroplasts and mitochondria. FEBS Journal 276: 3870–3880. 19523112 10.1111/j.1742-4658.2009.07102.x

[54] Buchanan-Wollaston V, Page T, Harrison E, Breeze E, Lim PO, et al (2005) Comparative transcriptome analysis reveals significant differences in gene expression and signalling pathways between developmental and dark/starvation-induced senescence in *Arabidopsis* . Plant J 42: 567–585. 15860015 10.1111/j.1365-313X.2005.02399.x

[55] Fujisawa Y, Kato T, Ohki S, Ishikawa A, Kitano H, et al (1999) Suppression of the heterotrimeric G protein causes abnormal morphology, including dwarfism, in rice. Proc Natl Acad Sci USA 96: 7575–7580. 10377457 10.1073/pnas.96.13.7575 PMC22128

[56] Trindade I, Capitao C, Dalmay T, Fevereiro MP, Santos DM (2009) miR398 and miR408 are up-regulated in response to water deficit in *Medicago truncatula* . Planta 231: 705–16. 20012085 10.1007/s00425-009-1078-0

[57] Sunkar R (2010) MicroRNAs with macro-effects on plant stress responses. Semin Cell Dev Biol 21: 805–811. 20398781 10.1016/j.semcdb.2010.04.001

[58] Yanik H, Türktaş M, Dundar E, Hernandez P, Dorado G, et al (2013) Genome-wide identification of alternate bearing-associated miRNA in the olive tree (*Olea europaea)* . BMC Plant Biol 13: 10. 23320600 10.1186/1471-2229-13-10 PMC3564680

[59] Khraiwesh B, Zhu JK, Zhu J (2012) Role of miRNAs and siRNAs in biotic and abiotic stress responses of plants. Biochim Biophys Acta 1819: 137–48. 21605713 10.1016/j.bbagrm.2011.05.001 PMC3175014

[60] Eldem V, Akcay CU, Ozhuner E, Bakir Y, Uranbey S, et al (2012) Genome-wide identification of miRNAs responsive to drought in peach (*Prunus persica*) by highthroughput deep sequencing. PLoS ONE 7(12): e50298. 23227166 10.1371/journal.pone.0050298 PMC3515591

[61] Ma L, Nie L, Liu J, Zhang B, Song S, et al (2012) An RNA-seq-based gene expression profiling of radiation-induced tumorigenic mammary epithelial cells. Genomics Proteomics Bioinformatics 10(6): 326–335. 23317700 10.1016/j.gpb.2012.11.001 PMC5054714

