# Supplementary material for: Boron Stress Responsive MicroRNAs and Their Targets in Barley
Source: PLoS One. 2013 Mar 26;8(3):e59543. doi: 10.1371/journal.pone.0059543 (PMC3608689; doi:10.1371/journal.pone.0059543)
Supplement: Figure S1 — The sequences, additional properties, and stem-loop secondary structure of pre-microRNAs of Hordeum vulgare (DOC) [file pone.0059543.s001.doc]

**Supplementary Fig. S1.** The sequences, additional properties, and stem-loop secondary structure of pre-microRNAs of *Hordeum vulgare*

UnigenCode: CL11872.Contig1

**miRNA name:** hvu-mir-159

**Pre-microRNA sequence:** GGUGGAGCUCCUAUCAUUCCAAUGAAGGGUCUACCGGAAGGGUUUGUGCAGCUGCUUGUUCAUGGUUCCCACUAUCCUAUCUCCAUUAGAACACGAGGAGAUAGGCUUGUGGUUUGCAUGAUCGAGGAGCCGCUUCGAUCCCUCGCUGACCGCUGUUUGGAUUGAAGGGAGCUCUGCA (178 bases)

**Mature sequence:** UUUGGAUUGAAGGGAGCUCUG (21 bases)

ΔG= -86.30

% GC content: 52.0

MEFI: **0.93**


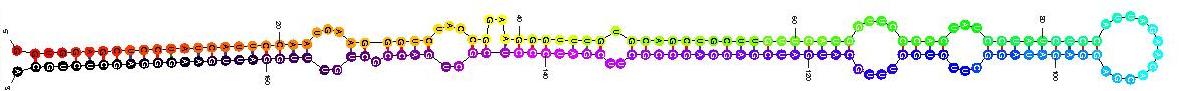


**Figure No: 1**

**UnigenCode:** CL25469.Contig1

**miRNA name:** hvu-mir-160

**Pre-microRNA sequence:** UGCGUGCCUGGCUCCCUGUAUGCCACUCAUGUAGCCCAACCCGCGGCGUGAUUGGAUGCUGUGGGUGGCGUGCAAGGAGCCAAGCAUGCGUACAUACA (98 bases)

**Mature sequence:** UGCCUGGCUCCCUGUAUGCCA (21 bases)


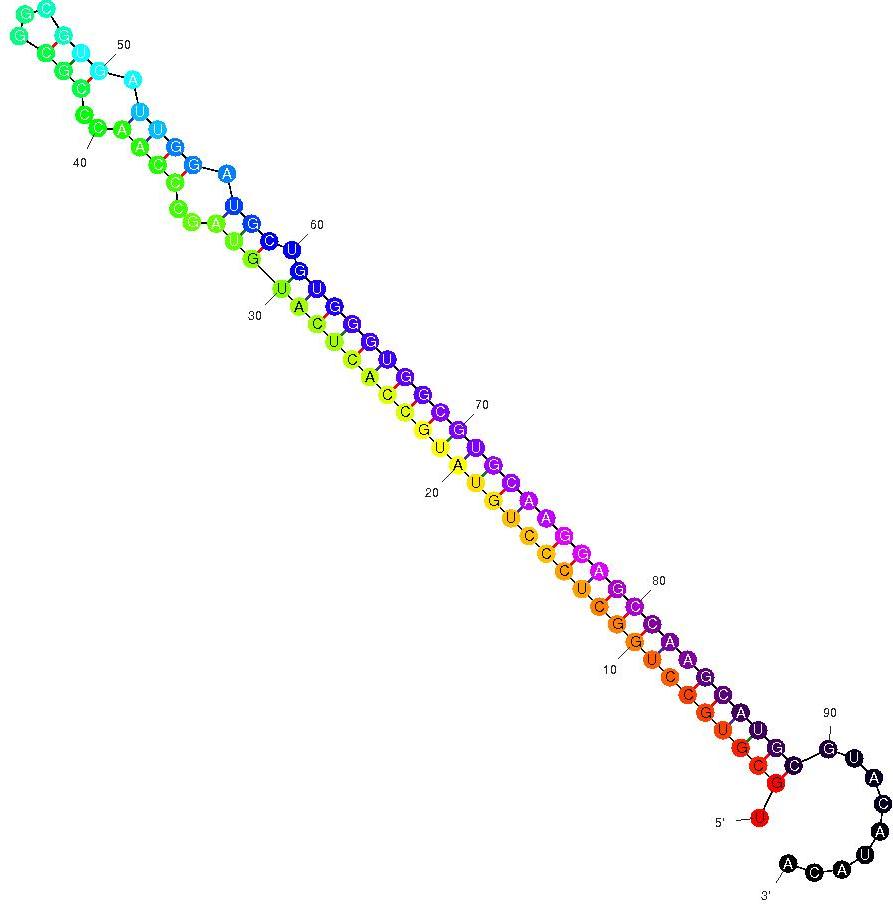
 ΔG= -56.00

% GC content: 60.0

MEFI: **0.95**

**Figure No: 2**

**UnigenCode:** CL11411.Contig1

**miRNA name:** hvu-mir-168

**Pre-microRNA sequence:** GUCCGUCGCCGCCGCCUCGGGCUCGCUUGGUGCAGAUCGGGACCCUCCGCCCGCCCCGACGGGCCGGAUCCCGCCUUGCACCAAGUGAAUCGGAGCCGGCGCAGCG (106 bases)

**Mature sequence:** GAUCCCGCCUUGCACCAAGUGAAU (24 bases)

ΔG= -64.40


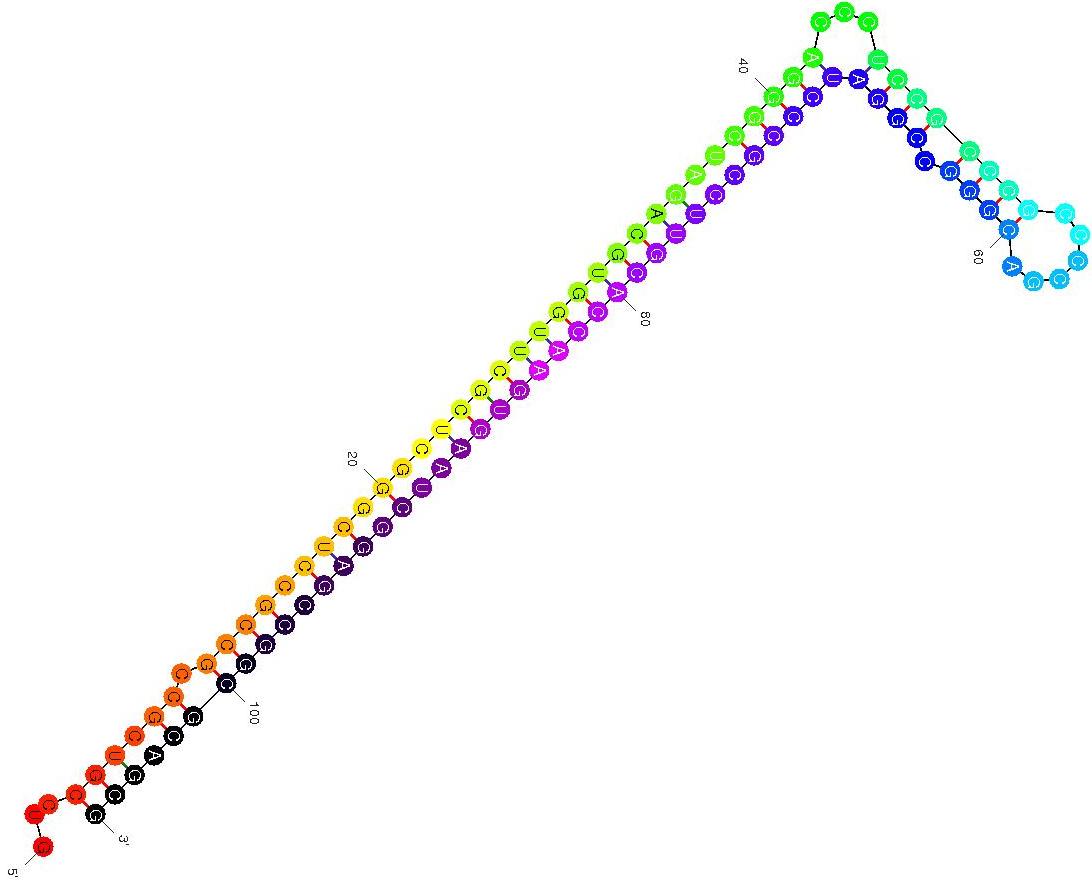
% GC content: 75.0

MEFI: **0.81**

**Figure No: 3**

**UnigenCode:** CL1724.Contig2

**miRNA name:** hvu-mir-171

**Pre-microRNA sequence:** GACAAGAGGAGGAUGCUGAAACGGUCACUAUGAUGUUGGCUCGACUCACUCAGACCACGCCGGAGGGAGCCAUCUGCGGCGGCGGUUCUGAUUGAGCCGUGCCAAUAUCUUAGUGCUCUUUCAUGCUCGUCCCUUGU (137 bases)

**Mature sequence:** UGAUUGAGCCGUGCCAAUAUC (21 bases)

ΔG= -73.20

% GC content: 55

MEFI: **0.97**

**
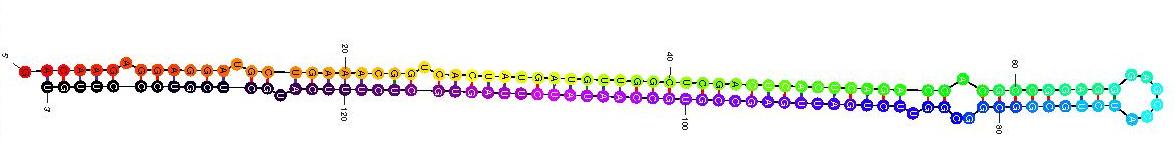
**

**Figure No: 4**

**UnigenCode:** CL16998.Contig1

**miRNA name:** hvu-mir-319a

**Pre-microRNA sequence:**

GGGAGCUCACUUCAGUCCACUCAUGGGAGGUAGCGGGGAUUGAACGAGCUGCCGAUUCAUUCACUCGAGCACACAGUGGAUACGAGACUUUAACAUCAGACCGCAAUAUUUACUGUGCGAAUGAGCGAAUGCAGCGGGAGAUUGUUCUCUCUUUCCUCCUCCAUGCUUGGACUGAAGGGAGCUCCC (186 bases)

**Mature sequence:** UUGGACUGAAGGGAGCUCCC (20 bases)

ΔG= -87.70

% GC content: 52

MEFI: **0.90**

**
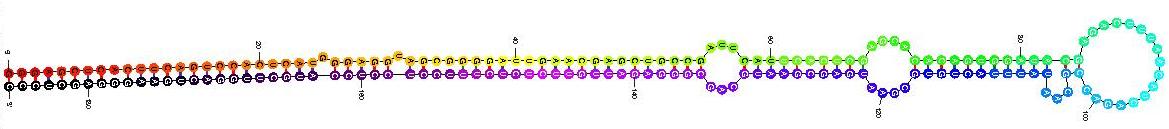
**

**Figure No: 5**

**UnigenCode:** CL26211.Contig1

**miRNA name:** hvu-mir-397

**Pre-microRNA sequence:**

CGCAGAGGUGCCGUUGAGUGCAGCGUUGAUGAACCGUCCGGCCAUGGCCCGUCCGCCUCCACCGAGGCCGGAGCGGUUCACCGGCGCUGCACGCAAUGACGCCUCUGCUUUCU

(133 bases)

**Mature sequence:** CCGUUGAGUGCAGCGUUGAUG (21 bases)

ΔG= -74.90


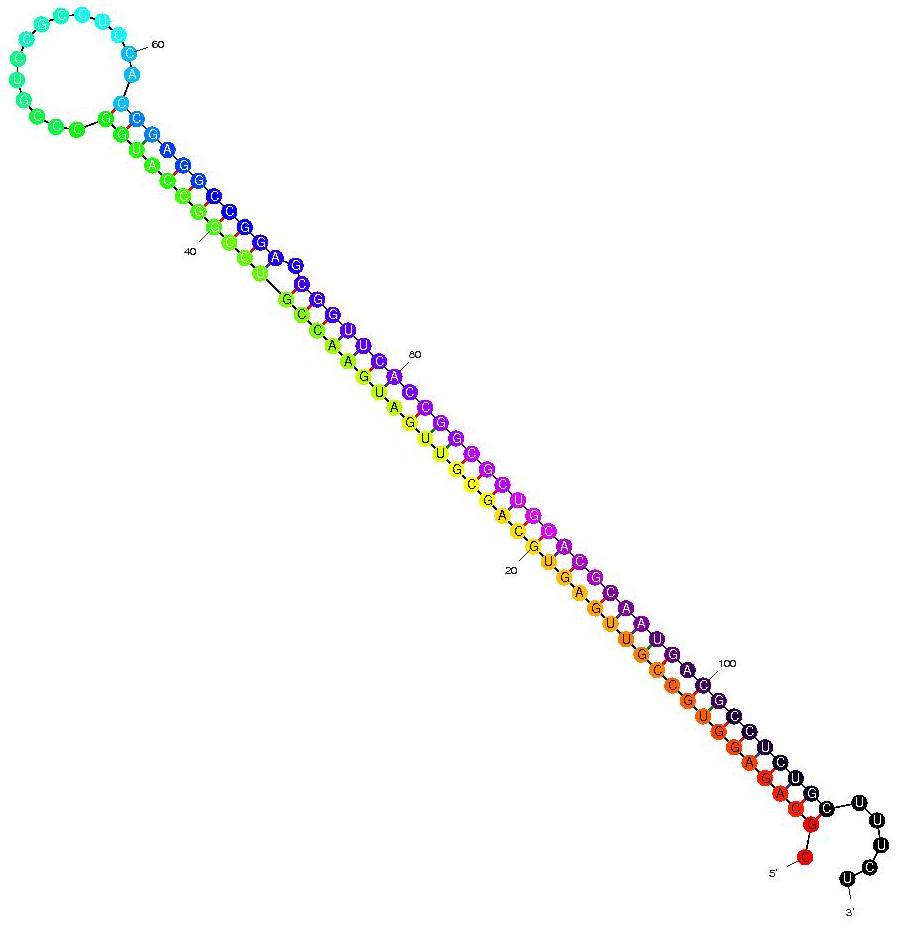
% GC content: 67

MEFI: **0. 98**

**Figure No: 6**

**UnigenCode:** CL951.Contig5

**miRNA name:** hvu-mir-444b

**Pre-microRNA sequence:**

GGUGGCACCAAGCAUGAGGCAACAACUGCAUUACUUUCAAGGAAGUUACAAAAUCUAUGGGUCUUCAUAAUCGUGACUUUCUUGCAAGUUGUGCAGUUGCUGUCUCAAGCUUGCUGACUCC (121 bases)

**Mature sequence:** UGCAGUUGCUGUCUCAAGCUU (21 bases)

ΔG= -55.20

% GC content: 45

MEFI: **1.01**

**
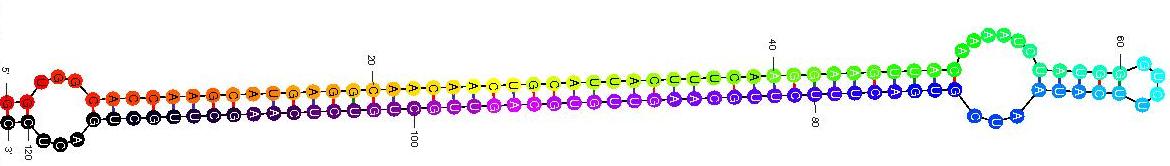
**

**Figure No: 7**

**UnigenCode:** CL16146.Contig1

**miRNA name:** hvu-mir-1120

**Pre-microRNA sequence:** CUACUCCCUCCUUCCCAUAUAUAAGAGUGUUUUUAAUACUACACUAGUGUGAAAAACAUUCUUAUAUUAUGGGACGGAGGAGUU (84 bases)

**Mature sequence:** ACAUUCUUAUAUUAUGGGACGGAG (24 bases)


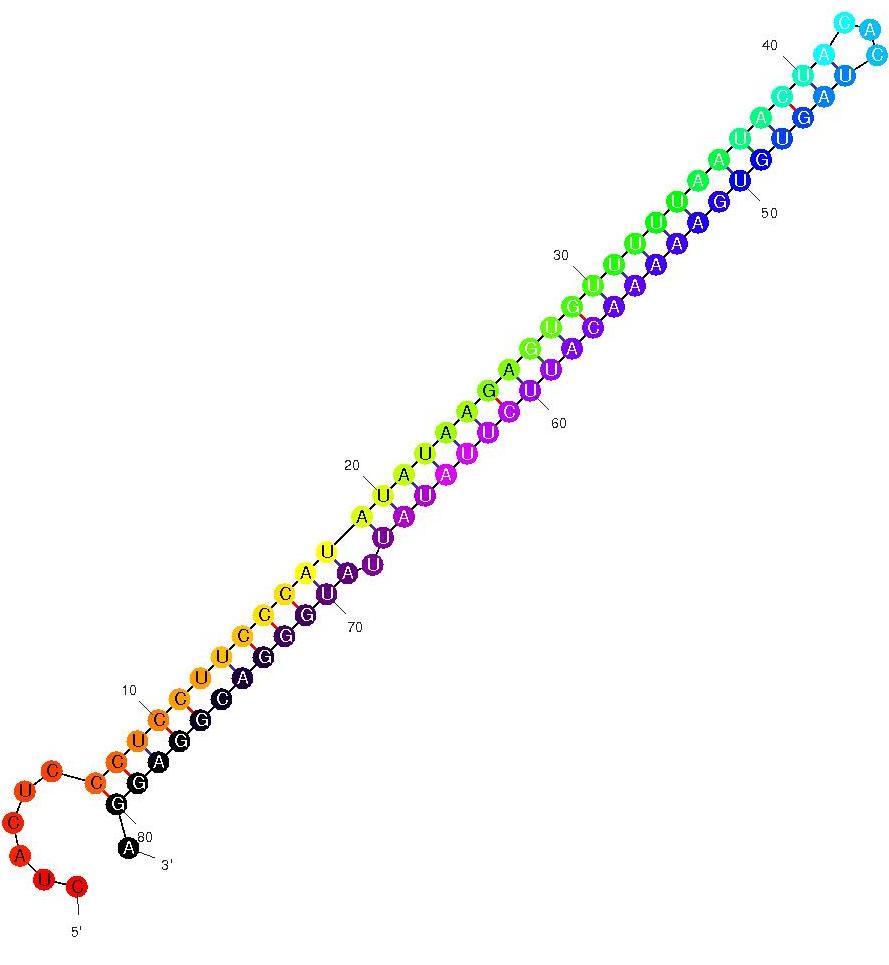
 ΔG= -41.30

% GC content: 36

MEFI: **1.36**

**Figure No: 8**

**UnigenCode:** CL63.Contig6

**miRNA name:** hvu-mir-1121

**Pre-microRNA sequence:** UCCCUCUGUAAAGAAAUAUAACAGUGUUUGGAUCACUAAAGUAGUGAUCUAAACGCUCUUAUAUUAGUUUACGGAGGGAGUAC (83 bases)

**Mature sequence:** AGUAGUGAUCUAAACGCUCUUA (22 bases)

ΔG= -45.90

% GC content: 36

MEFI: **1.53 (best ever!)**

**
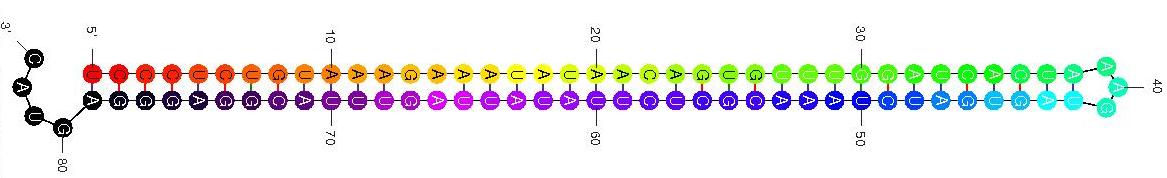
**

**Figure No: 9**

**UnigenCode:** CL86.Contig7

**miRNA name:** hvu-mir-2004

**Pre-microRNA sequence:** GAAUGUGUUUGUUUUUAUGUUAUUUUGUGAAGGAAUGAAAACAAUUCUAUAUAGAAUGGCUUGGGAAUUAUGCCUAGA (78 bases)

**Mature sequence:** UUUGUUUUUAUGUUAUUUUGUGAA (24 bases)

ΔG= -16.90


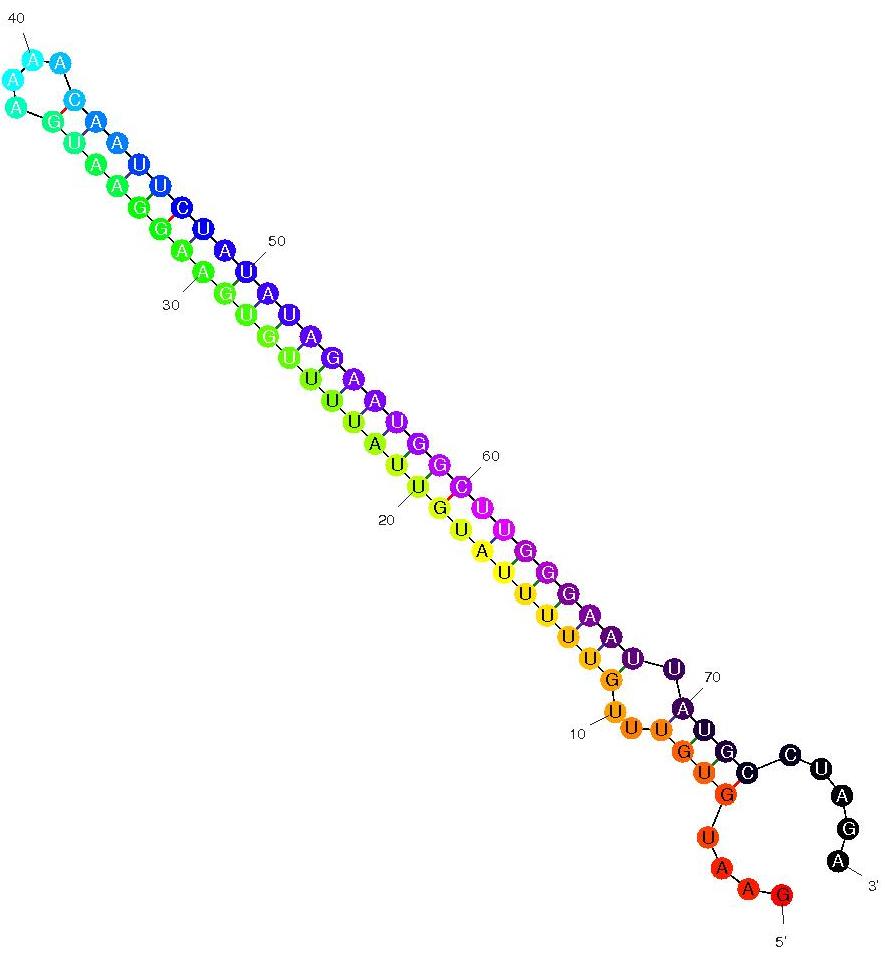
% GC content: 29

MEFI: **0.74**

**Figure No: 10**

**UnigenCode:** CL351.Contig1

**miRNA name:** hvu-mir-2007

**Pre-microRNA sequence:** AGAUUCAAAAUACCCAAUAUCUUGCUAGAACAAGAUAUUGGGUAUUUUUGUCUU (54 bases)

**Mature sequence:** CAAGAUAUUGGGUAUUUUUGUC (22 bases)

**ΔG**= -25.90

**% GC content**: 30

**MEFI**: **1.59**

**
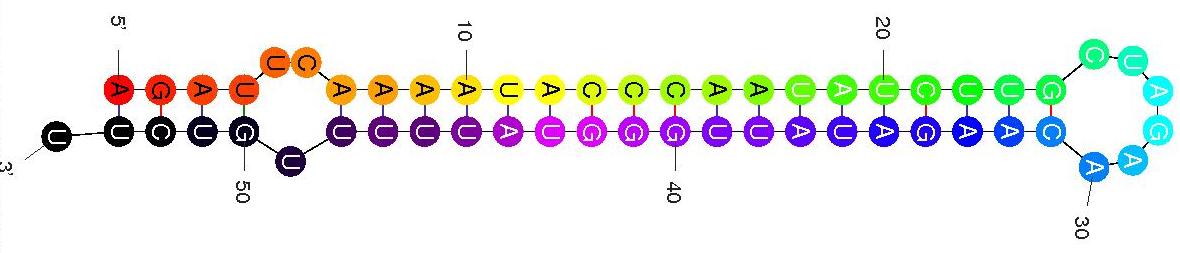
**

**Figure No: 11**

**UnigenCode:** CL10125.Contig1

**miRNA name:** hvu-mir-2024a

**Pre-microRNA sequence:**

GGCACCAAGCAUGAGGCAACAACUGCAUUACUUUCAAGGAAGUUACAAAAUCUAUGGGUCUUCAUAAUCGUGACUUUCUUGCAAGUUGUGCAGUUGCUGUCUCAAGCUUGCUGACU

CC (118 bases)

**Mature sequence:** GCAGUUGCUGUCUCAAGCUU (20 bases)

**ΔG**= -53.40

**% GC content**: 44

**MEFI**: **1.02**

**
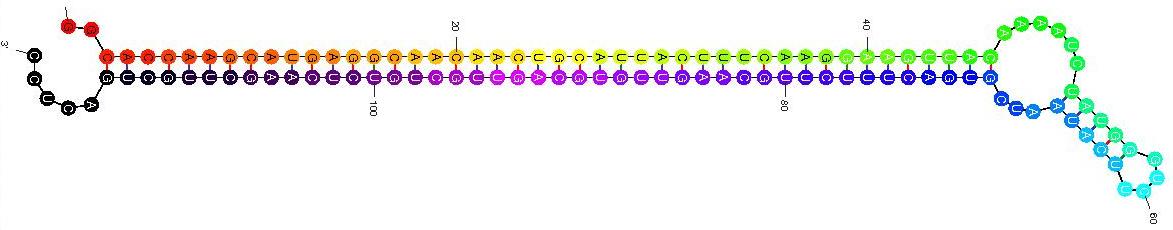
**

**Figure No: 12**

**UnigenCode:** CL26929.Contig1

**miRNA name:** hvu-mir-2906

**Pre-microRNA sequence:**

GGUGGUGUGGACGUUGCCGUAGCGCUGUGGGCCUGGCUCGGUCGUGCUUCUGCUGGCCCGCCCGUUCCAAGUUGCGUAGUGGACCCGGUUGGGGCCUUUGCGGAAGCUAGGCGUCACGGGCUAUAAUGUGGCAGGCAUUGCGUGAGGCUGGUUUCACAGAGCAGCGAAAACUGCCCGCUUCCAACGGUGGAAGGAUAACGGGCCGCUGCACAACUGGGCCGCUUGGGUCUCACAGCCUACUCCAACGAACCACC (254 bases)

**Mature sequence:** AACGGGCCGCUGCACAACUGG (21 bases)

**ΔG**= - 123.9

**% GC content**: 63


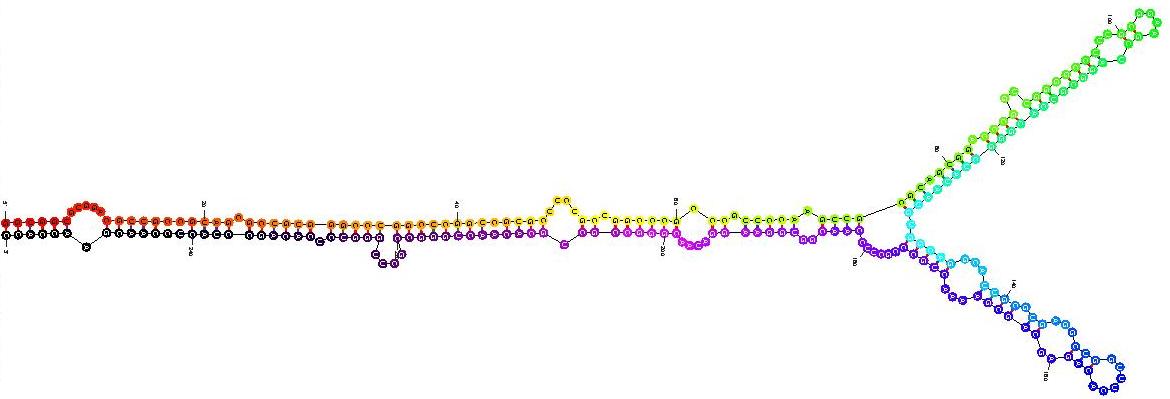
**MEFI**: **0.77**

**Figure No: 13**

**UnigenCode:** CL18.Contig22

**miRNA name:** hvu-mir-5049

**Pre-microRNA sequence:** ACUCCCUCUGUUCCUAAAUACUUGUUGUUGGGGAGAACUAGACUAGUUUUCUGCAACAACAAGUAUUGUGGUACGGAGGGA (81 bases)

**Mature sequence:** UCCUAAAUACUUGUUGUUGGG (21 bases)

**ΔG**= -47.80

**% GC content**: 43

**MEFI**: **1.37**

**
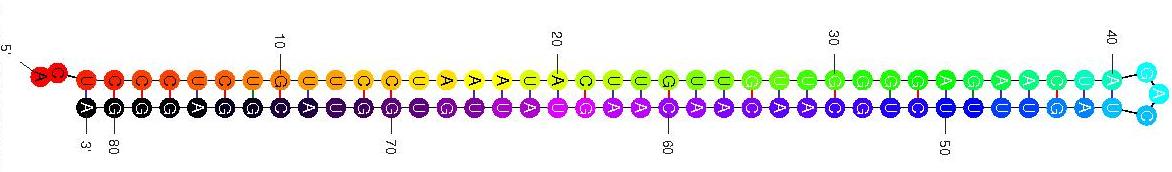
**

**Figure No: 14**

**UnigenCode:** CL16658.Contig1

**miRNA name:** hvu-mir-5051

**Pre-microRNA sequence:** GCAGGUACUGUUCCAGUUUCAAGGUUUCAAAGCUGAAGGCUUCCCAGAGAUCCUGCUGCUGGGUGGUCUUUAGCUUUGGCACCUUGAAACUGGGACUUUAGCUUU (105 bases)

**Mature sequence:** UUUGGCACCUUGAAACUGGGA (21 bases)


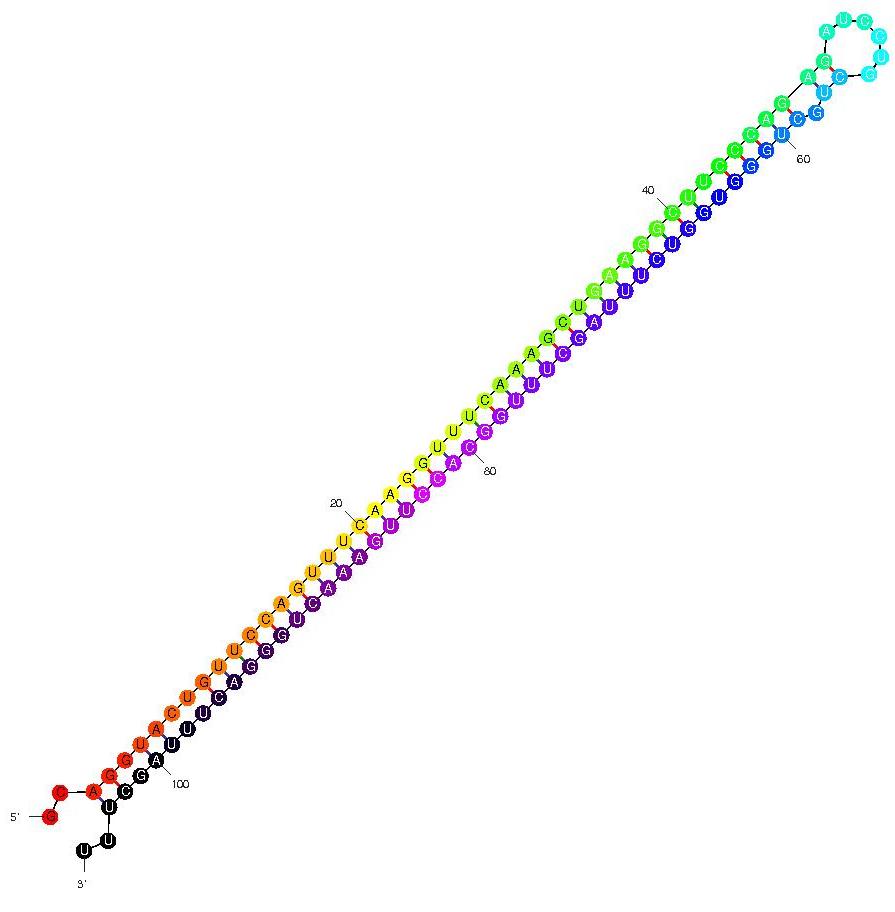
 **ΔG**= -61.90

**% GC content**: 49

**MEFI**: **1.20**

**Figure No: 15**

**UnigenCode:** CL26929.Contig1

**miRNA name:** hvu-mir-2906

**Pre-microRNA sequence:**

GGUGGUGUGGACGUUGCCGUAGCGCUGUGGGCCUGGCUCGGUCGUGCUUCUGCUGGCCCGCCCGUUCCAAGUUGCGUAGUGGACCCGGUUGGGGCCUUUGCGGAAGCUAGGCGUCACGGGCUAUAAUGUGGCAGGCAUUGCGUGAGGCUGGUUUCACAGAGCAGCGAAAACUGCCCGCUUCCAACGGUGGAAGGAUAACGGGCCGCUGCACAACUGGGCCGCUUGGGUCUCACAGCCUACUCCAACGAACCACC (254 bases)

**Mature sequence:** AACGGGCCGCUGCACAACUGG (21 bases)

**ΔG**= - 123.9

**% GC content**: 63


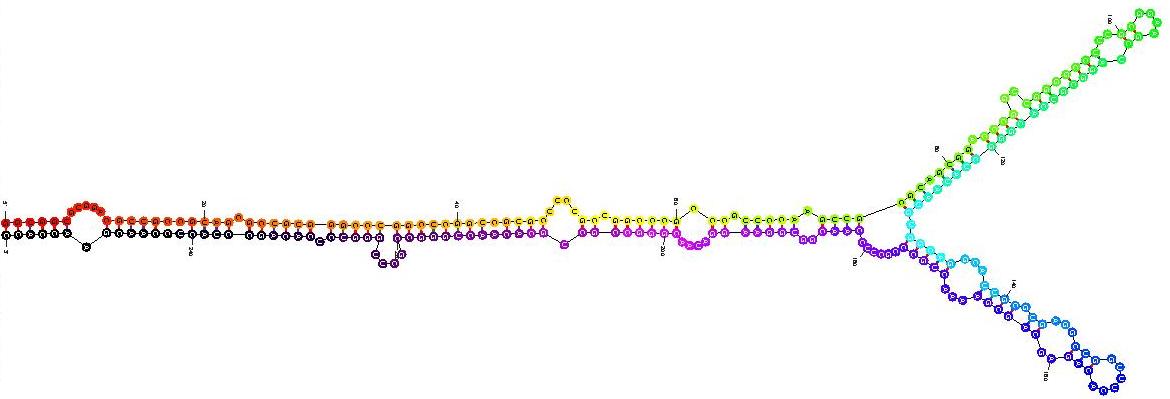
**MEFI**: **0.77**

**Figure No: 16**

**UnigenCode:** CL14337.Contig1

**miRNA name:** hvu-mir-157

**Pre-microRNA sequence:** UUGACAGAAGAGAGUGAGCACACGGCGUGAUGCCGGCAUAACAUGUAUGCCGUCUUCGCCGCGUGCUCACUCCUCUUUCUGUCAG(85 bases)

**Mature sequence:** UUGACAGAAGAGAGUGAGCAC (21 bases)

ΔG= -52.40

% GC content: 55.0

MEFI: **1.12**

**
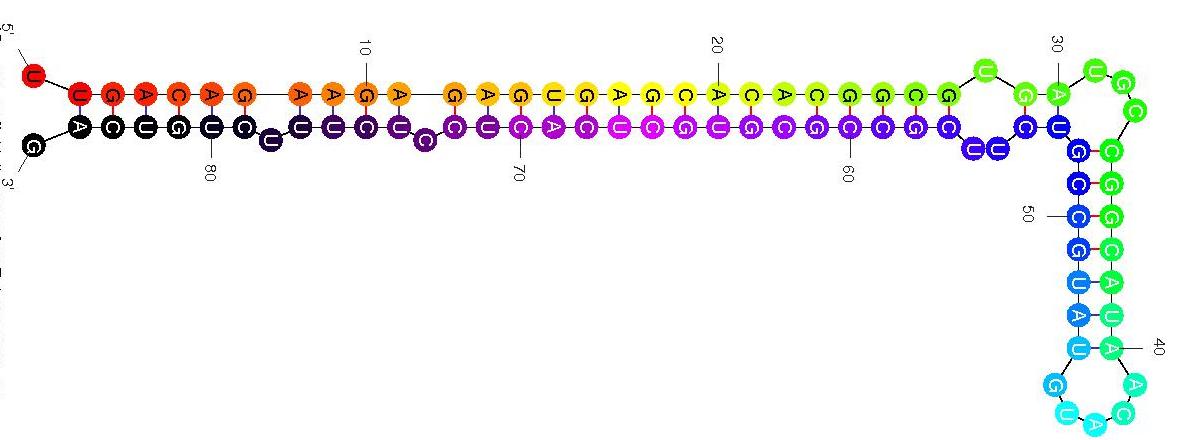
**

**Figure No: 17**

**UnigenCode:** CL5590.Contig1

**miRNA name:** hvu-mir-169

**Pre-microRNA sequence:** UAACACGCCAGAAAUAAAGCCAAGGAUGAGUUGCCUGAUCAUCAGGACUCAUGGAGGUAGGGCUUUCUGUUUUUGGUGAGUUU (83 bases)

**Mature sequence:** AAGCCAAGGAUGAGUUGCCUG (21 bases)

ΔG= -30.10

% GC content: 45.0

MEFI: **0.80**

**
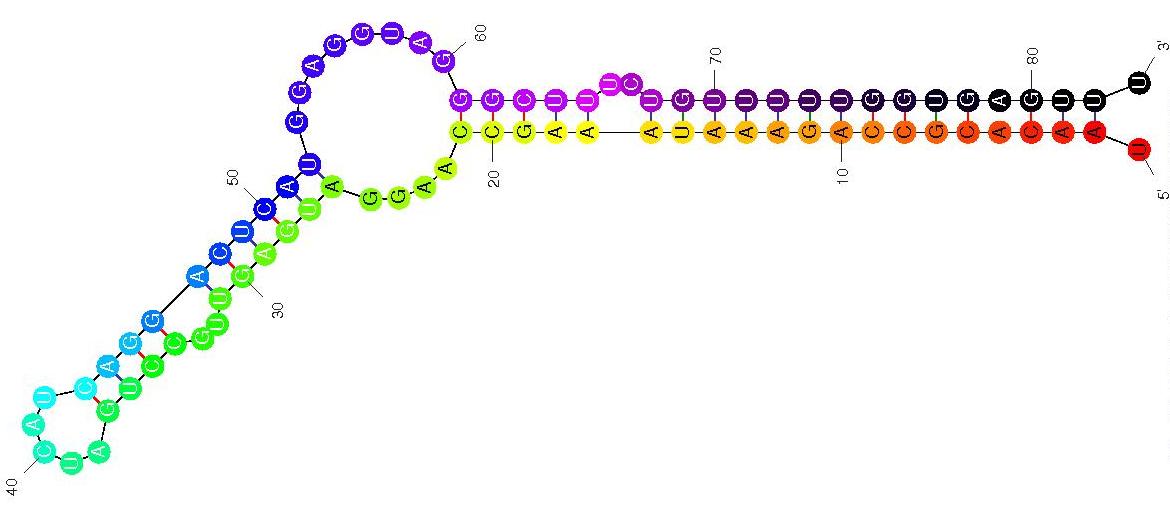
**

**Figure No: 18**

**UnigenCode:** CL56.Contig14

**miRNA name:** hvu-mir-1122 and hvu-mir-1126

**Pre-microRNA sequence:** AGUCCUUUUAGAGAUUCCACUAUGGACUACAUACGGAGCAAAAUGAGUCAAUCUACACUCUAAAGUAUAUCUUUGUACAUCCGUAUGUAGUUUGUAGUGUAAUGUCUAAAAAGACAUAUA (120 bases)

**Mature sequence:** UUUGUACAUCCGUAUGUAGU (mir-122 20 bases)

**Notice that** potantially mir-1122 and mir-1126 should be miStar (*) sequence of each other. In order to clarify ambiguity, we will definitely determine the exact subchoromosomal location of them.

UCCACUAUGGACUACAUACGGAG (mir-126 23 bases)


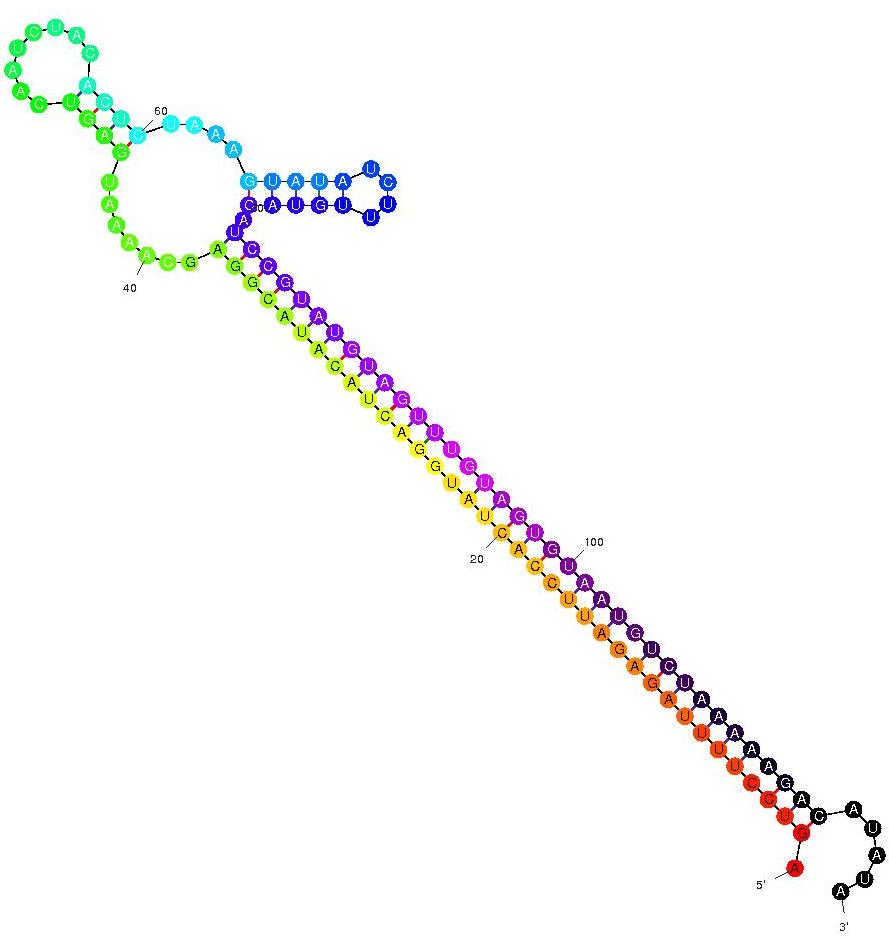
 ΔG= -50.70

% GC content: 33

MEFI: **1.28**

**Figure No: 19**

**UnigenCode:** CL10125.Contig1

**miRNA name:** hvu-mir-2023a

**Pre-microRNA sequence:**

GCCGGUGUUUUUGCCGGUUGAACGACCUCACCAUGUCGACCGCCUCUUCCUUGACAGGUACCAGGAAGAAAAGAUCUGCUCGUGCCAUGUCGAGGUCGUUCGACCAGCAAAAC

(113 bases)

**Mature sequence:** UUUUGCCGGUUGAACGACCUCA (22 bases)

**ΔG**= -46.00

**% GC content**: 55

**MEFI**: **0.74**

**
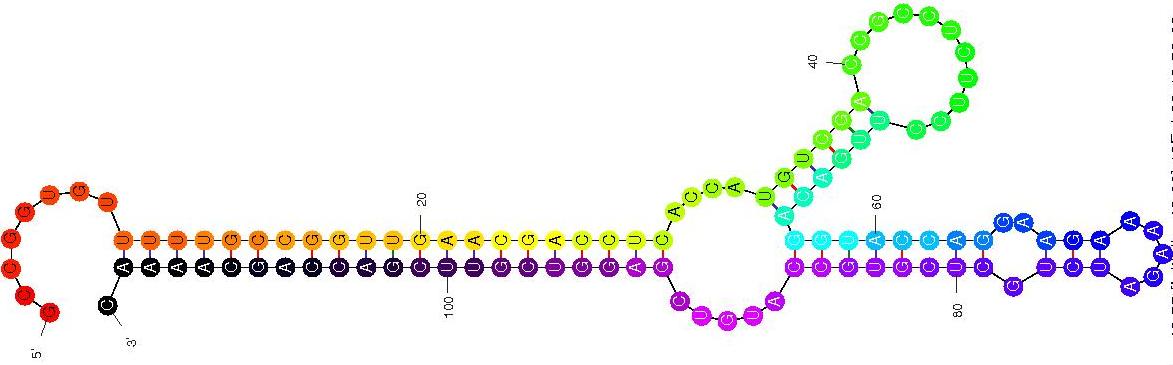
**

**Figure No: 20**

**UnigenCode:** CL8171.Contig1

**miRNA name:** hvu-mir-5048

**Pre-microRNA sequence:** CUAGCAAUAUAUUUGCAGGUUUUAGGUCUAAGUGGUGAAUAUCGAUCUCUGAAUAUAUUGAUAACCUUCACAAAUUUGCUGAUUAUUUUGUAGAGCUAUUUCAUGAGAAAUGAUUGUCCCACAUGCAUAUCCAUGACUAAAAAAAGCAAAAAAGUAAUAAUUGGGUGUUGUGUUCUCACAAAGAAAGAUGGAAAAGUUUAACAAAAACUUGUGAAGGAAGAUGAAUGCUUUGAUUAUGCAUGAAUCCAUAAUGAUAAAGUGCUAUUGAAUGAUCAAGUAAUUUGUAAGGUUUUUAUUAUCUUCGCAAGAGUUGUAUUGUUAAUUUAGACCUAGACAUGCAAGUAUAUUUCUAGG (354 bases)

**Mature sequence:** UAUUUGCAGGUUUUAGGUCUAA (22 bases)

**ΔG**= -96.90

**% GC content**: 31

**MEFI**: **0.88**

**
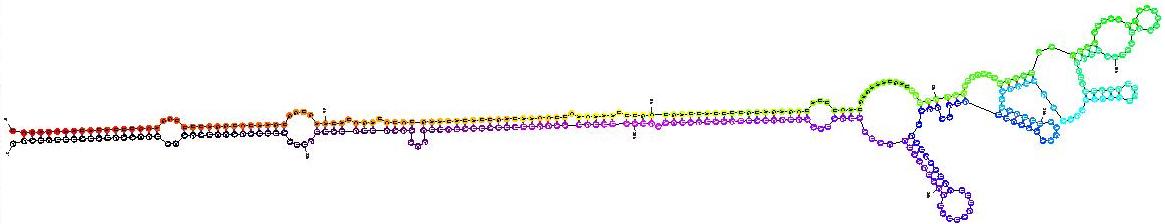
**

**Figure No: 21**

**UnigenCode:** CL13226.Contig1

**miRNA name:** hvu-mir-156

**Pre-microRNA sequence:**

UGACAGAAGAGAGAGAGCACAGUCGGAGUCGAGCACGUGCGUCAACUGUCCAUCGGAGAACAUCUUGUUGCUGCUGCUGCUCUCGGGCGGCGGCGGCAGCGGGGCGACGGCCUUGAGGAGAGGCUGGCACACGGGGAUCUCGAGUGCCGGCGCGCCGGUGCUGAAGCUCAUCUGGUCU (178 bases)

**Mature sequence:** UGACAGAAGAGAGAGAGCAC (20 bases)

ΔG= -83.20


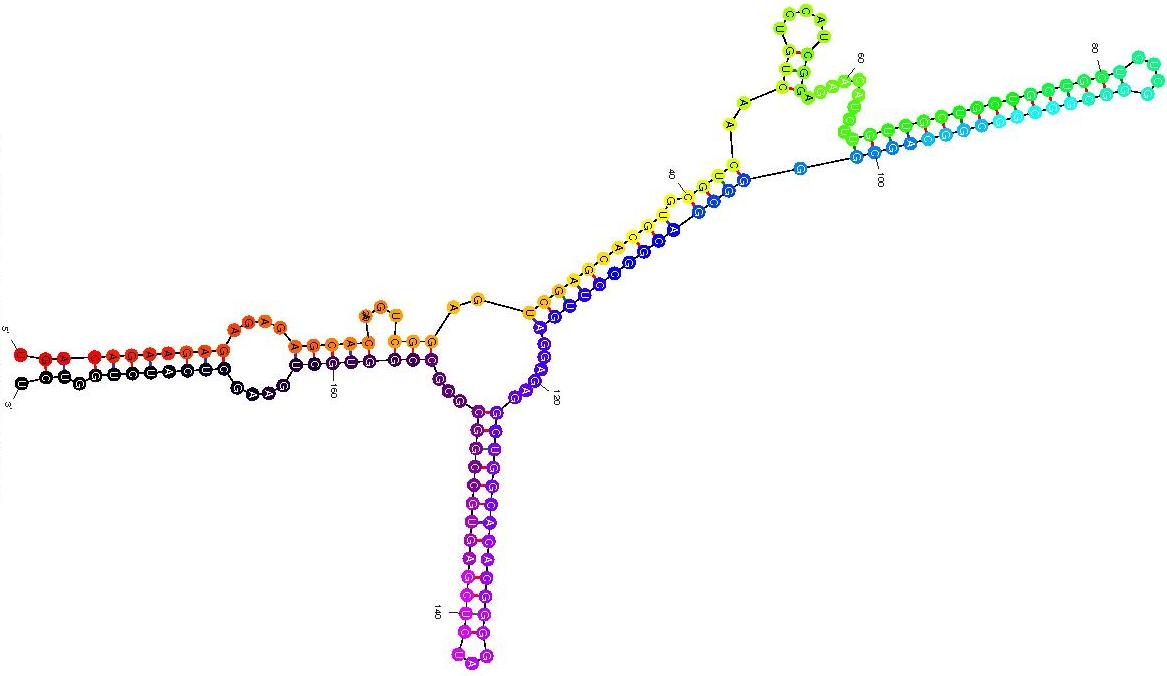
% GC content: 65.0

MEFI: **0.71**

**Figure No: 22**

**UnigenCode:** CL10723.Contig2

**miRNA name:** hvu-mir-408

**Pre-microRNA sequence:**

GGAGACAGGGAUGGAGCAGAGCAAGGGAUGGGGCAAGCAACAAACUAUCACCCCCUUAUCAUGAGAAGAUCGAGAGAGUUGUGAGAGACCAGGGAUCCCUGUCGUCGUUGUUGUUCCUCCCUCCCUGCACUGCCUCUUCCCUGGCUCCC (149 bases)

**Mature sequence:** CUGCACUGCCUCUUCCCUGGC (21 bases)

ΔG= -67.50

% GC content: 56

MEFI: **0.80**

**
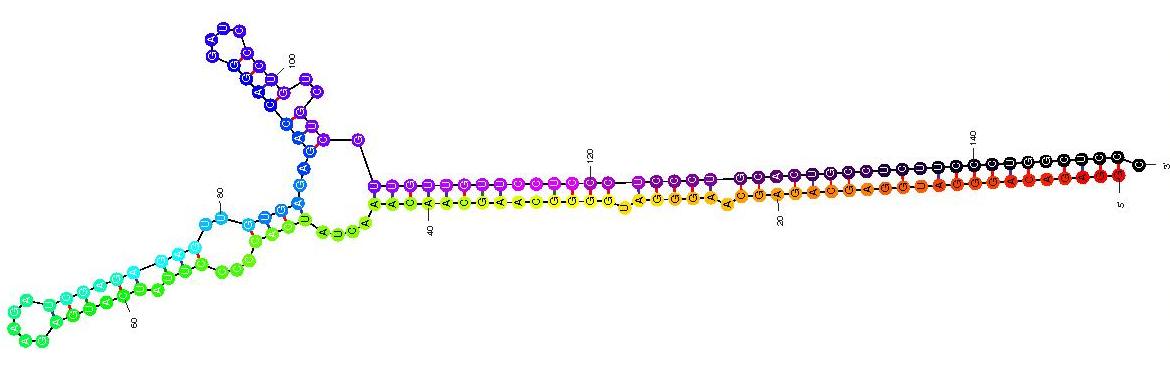
**

**Figure No: 23**

**UnigenCode:** Unigene28555

**miRNA name:** hvu-mir-5052

**Pre-microRNA sequence:** UUCUACCAUAACCGGCUGGACGGUAGGCAUACACAUCCUACCGCCACAAGCAGUAACUGUAGGGCUCGGAUCAAUGCGGAUGGUUCCUUCGGGUCAACGGAGUCAAACGACCUUAUCGCCAUUGUCUGCAGCGAUAGGAUGUGUAUGCCUACCGCCAUAGCGGGUGACGGUAGGA (175 bases)

**Mature sequence:** ACCGGCUGGACGGUAGGCAUA (21 bases)

**ΔG**= -85.00

**% GC content**: 54


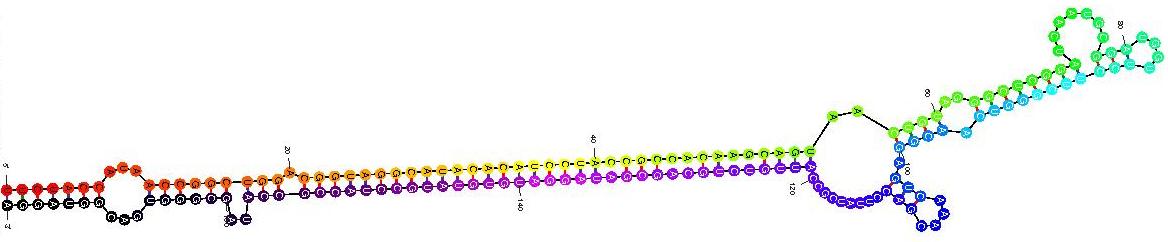
**MEFI**: **0.89**

**Figure No: 24**

**UnigenCode:** CL1686.Contig2

**miRNA name:** hvu-mir-164

**Pre-microRNA sequence:** GCUGCUGUUGCUGGCACUGUUAUUACUGAAGCUGGAGAAGCAGGGCACUUGCUCAAGUGCGGCCGUGGCGGCGGC (75 bases)

**Mature sequence:** UGGAGAAGCAGGGCACUUGCU (21 bases)

ΔG= -34.10

% GC content: 61.0

MEFI: **0.74**

**
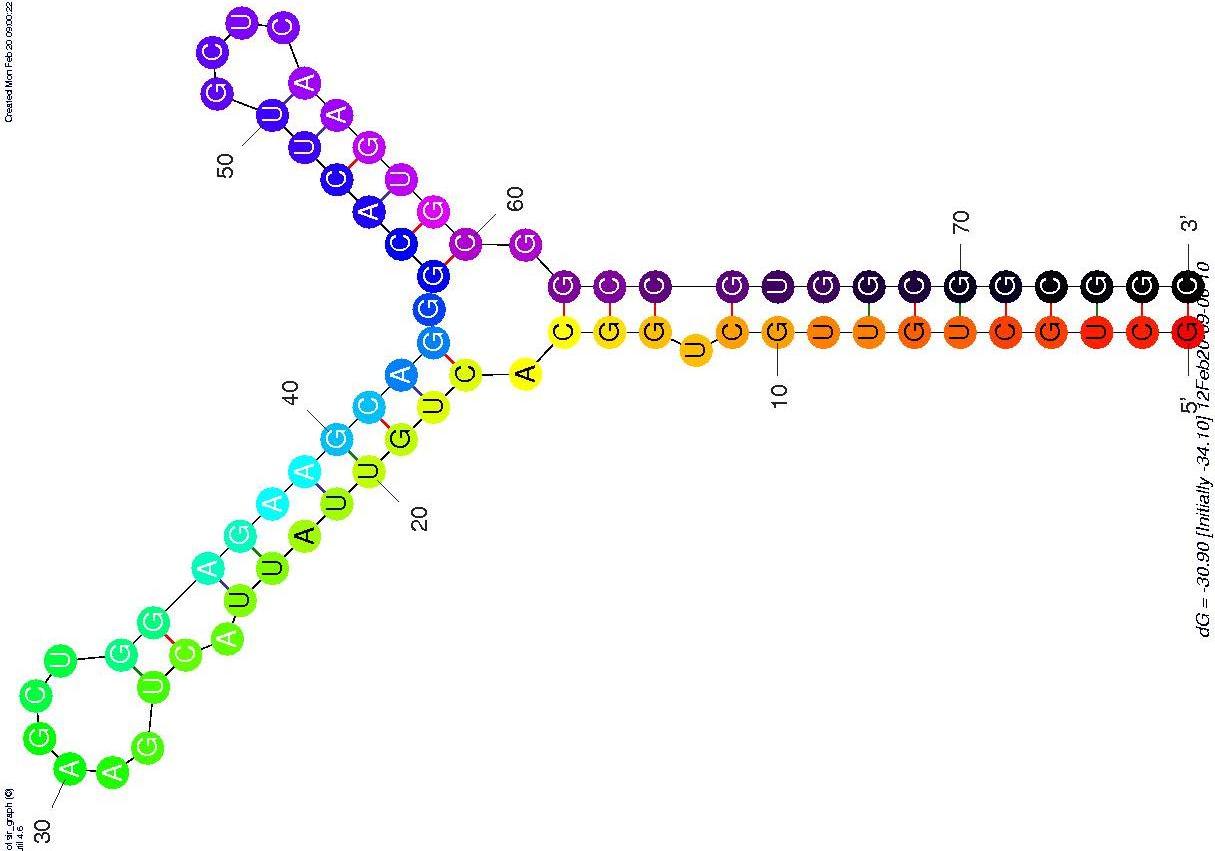
**

**Figure No: 25**

**UnigenCode:** CL876.Contig4

**miRNA name:** hvu-mir-399

**Pre-microRNA sequence:**

UAGGUAGAUUUGCCAAAGGAGAUUUGCCGAACUAUAGGUACUGUAGGUAGGUUUUGCCAAAAGGAGAUUUGCCCCGAACUAUCGGUAGGUUUGCCAAAGGAGAUUUGCCCCGA

(113 bases)

**Mature sequence:** UGCCAAAGGAGAUUUGCCCCG (21 bases)

ΔG= -34.20

% GC content: 46


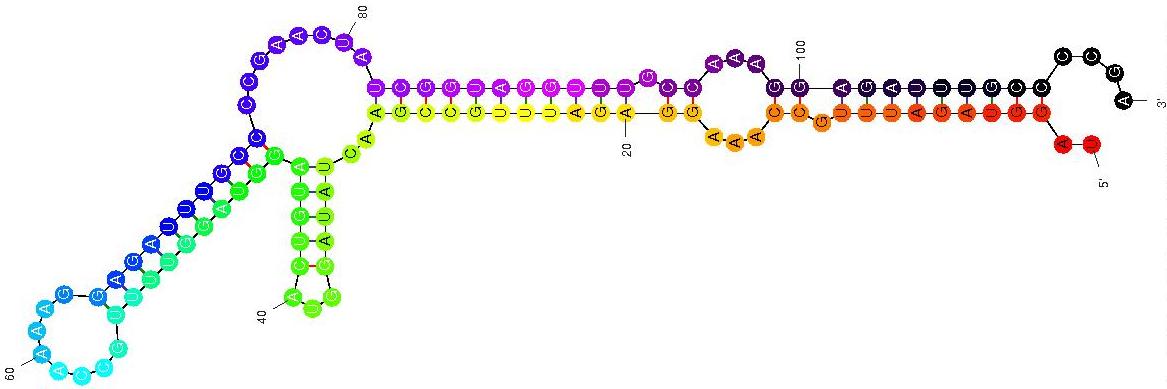
MEFI: **0.65**

**Figure No: 26**

**UnigenCode:** CL45161.Contig1

**miRNA name:** hvu-mir-2014

**Pre-microRNA sequence:** UGUUUUUGUGCCGUGAUGAUUGCUUUAUUUCUGACACCUCUUGUACGAGAAGCGCCUUGAUGCGCUUCGCUGAGCAGAUCGCAGCAAACAUAUCUGAGCACAGCCACAC (109 bases)

**Mature sequence:** AGCAAACAUAUCUGAGCACA (22 bases)

**ΔG**= -32.20

**% GC content**: 49

**MEFI**: **0.60**

**
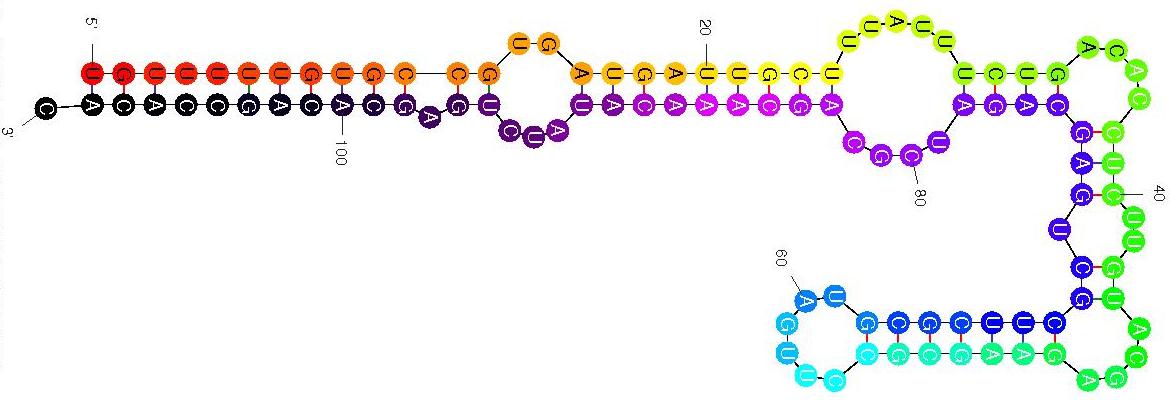
**

**Figure No: 27**

**UnigenCode:** CL8337.Contig1

**miRNA name:** hvu-mir-2914

**Pre-microRNA sequence:** UUCUGCCCUAUCAACUUUCGAUGGUAGGAUAGGGGCCUACCAUGGUGGUGACGGGUGACGGAG (63 bases)

**Mature sequence:** CAUGGUGGUGACGGGUGACGGAG (23 bases)

**ΔG**= -21.8


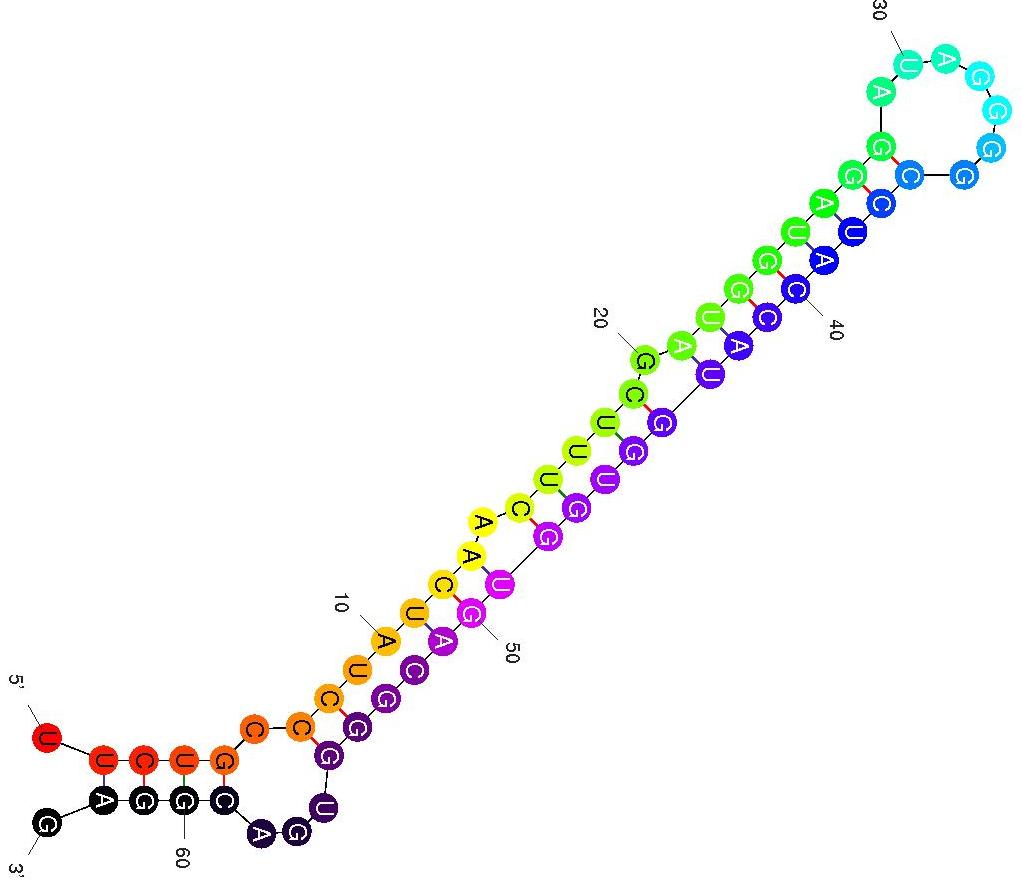
**% GC content**: 56

**MEFI**: **0.61**

**Figure No: 28**

**UnigenCode:** CL2600.Contig2

**miRNA name:** hvu-mir-165 (its pre-miRNAs is identical with *Sorghum bicolor* exept a mismatch the last nucleotides of mature miRNAs between them)

**Pre-microRNA sequence:** CCGCGACUGCCCCAUCCUCUGCAACAUCGAGCCCAAGCAGAUCAAGGUCUGGUUCCAGAACCGCAGAUGCCGCGAGAAGCAACGGAAGGAGGCCUCUCGC (100 bases)

**Mature sequence:** CCGCGACUGCCCCAUCCUCA (20 bases)

ΔG= -31.90

% GC content: 62.0


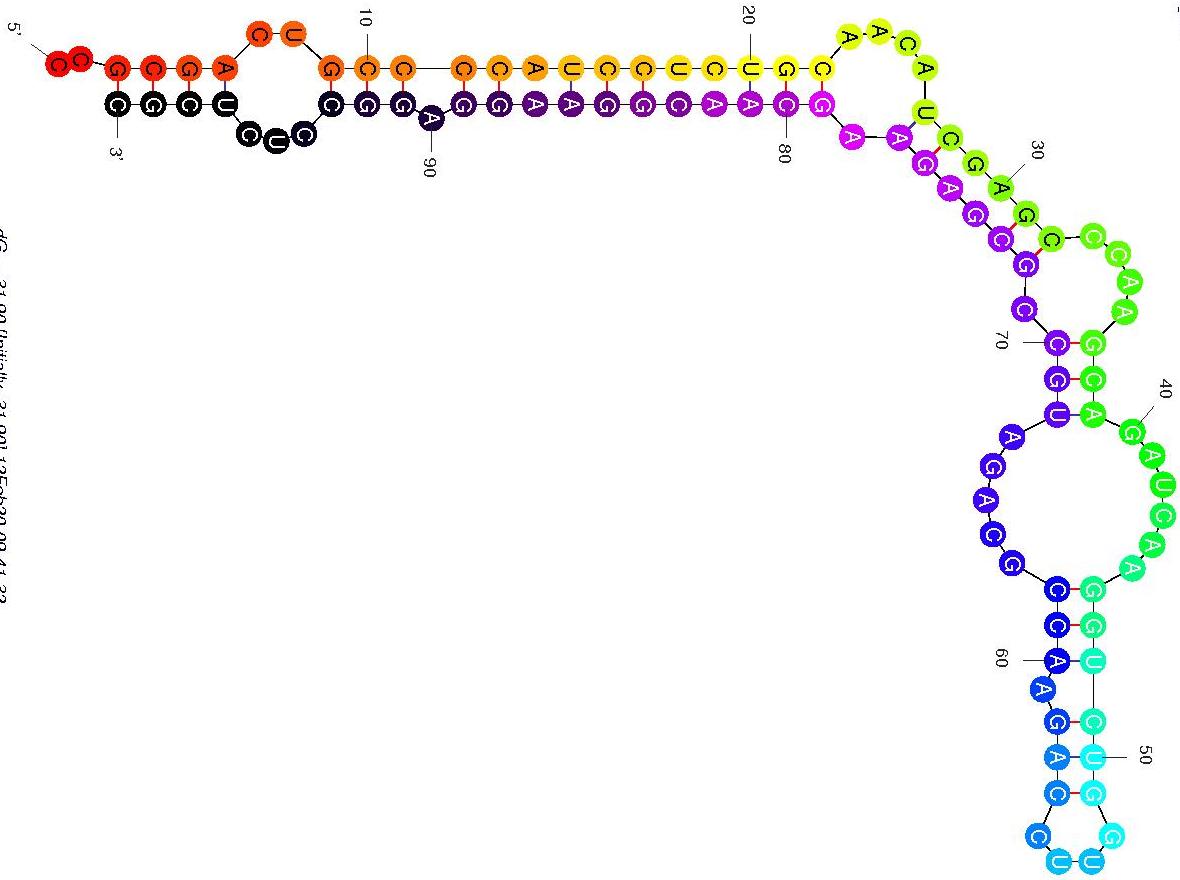
MEFI: **0.51**

**Figure No: 29**

**UnigenCode:** CL15989.Contig1

**miRNA name:** hvu-mir-172c

**Pre-microRNA sequence:** AGGAUCUUGAUGAUGCUGCUGAGAAGGCUGUAAAAGCAUCGGUCAAAUGAUUAA (54 bases)

**Mature sequence:** AGGAUCUUGAUGAUGCUGCUG (21 bases)

ΔG= -13.40

% GC content: 41

MEFI: **0.60**

**
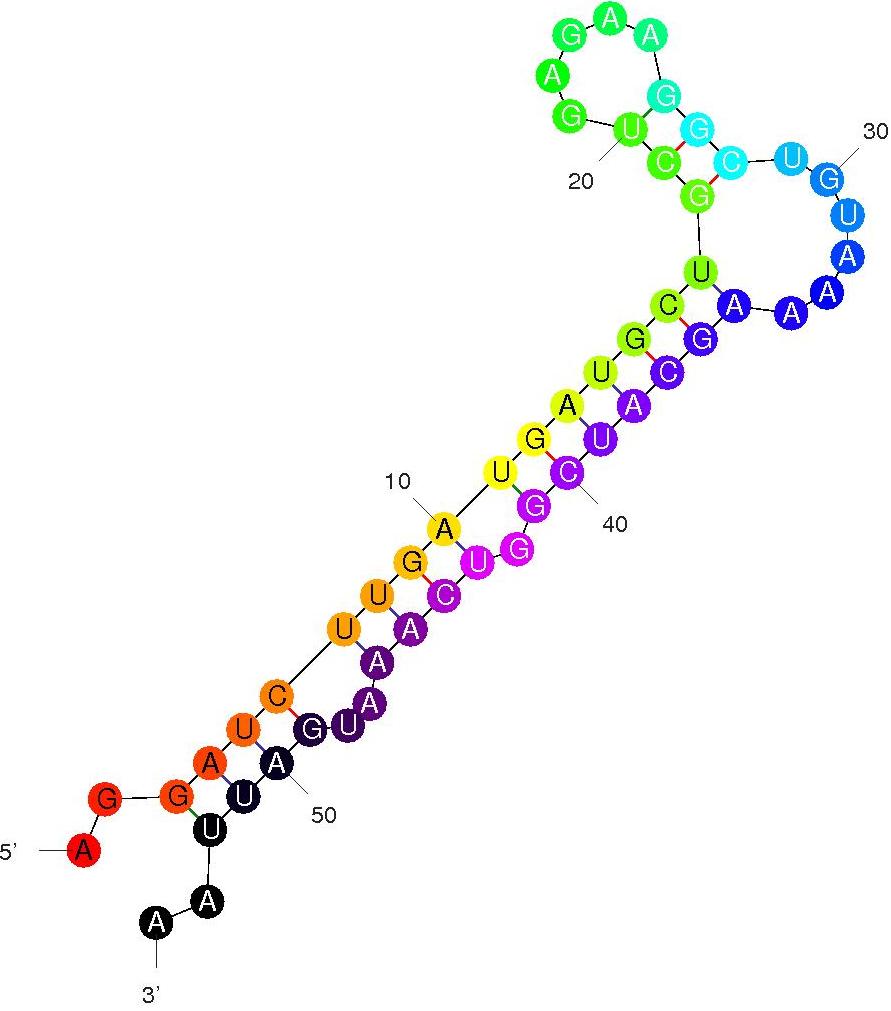
**

**Figure No: 30**

**UnigenCode:** CL2226.Contig1_All

**miRNA name:** hvu-mir-319c

**Pre-microRNA sequence:** UCCAGCAUAUUGGAAUGAAGGGAGCUCAAUCUUCCAAGGACCAGUUAUGGGCCUAGAAGUAGAGAAGUUGCCAUUUGA (78 bases)

**Mature sequence:** UUGGAAUGAAGGGAGCUCAA (20 bases)

ΔG= -19.60

% GC content: 45

MEFI: **0. 55**

**
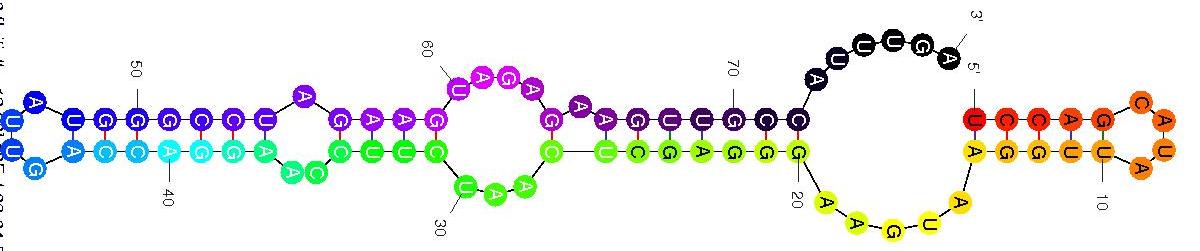
**

**Figure No: 31**

**UnigenCode:** CL258.Contig7

**miRNA name:** hvu-mir-2019

**Pre-microRNA sequence:** UGCAAAGUGUGCCGGAGCUGGCCAAGGCGUGCACCACCAUCAUCUGGAUCGGGUCGGCGCUGCAUGCGGCA (71 bases)

**Mature sequence:** CGGGUCGGCGCUGCAUGCGGC (21 bases)


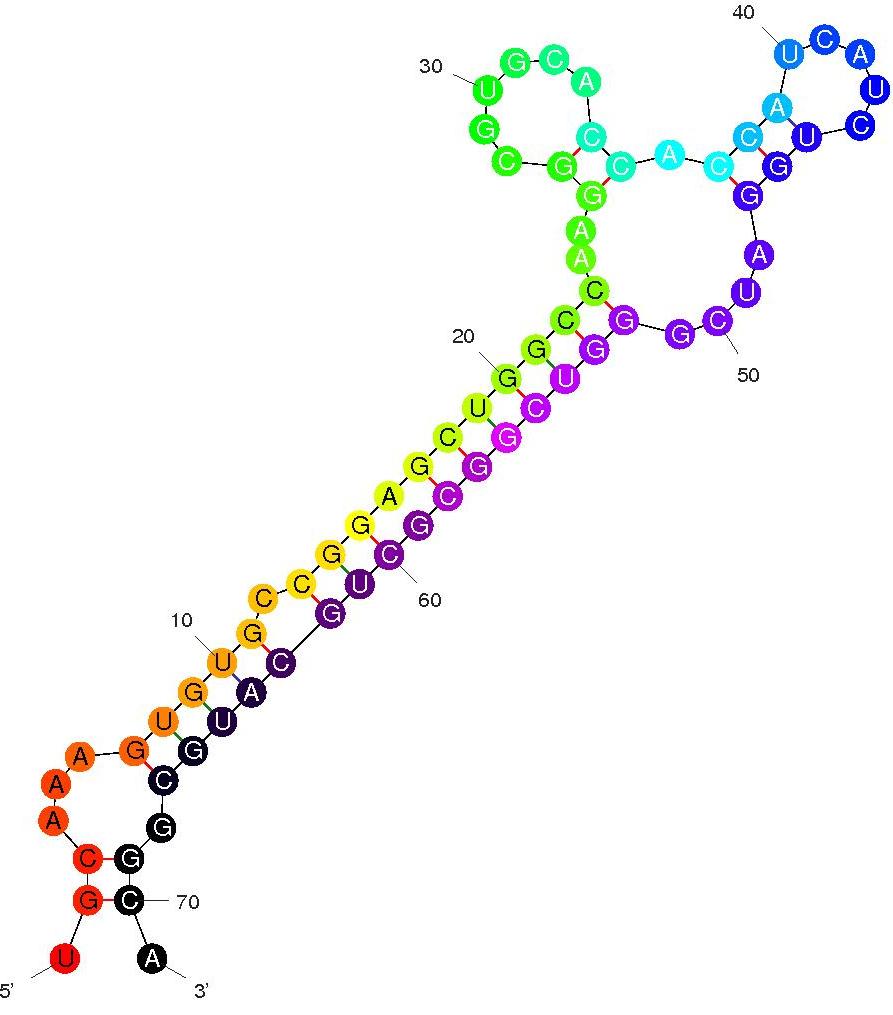
 **ΔG**= -24.70

**% GC content**: 65

**MEFI**: **0.53**

**Figure No: 32**

**UnigenCode:** CL8337.Contig1

**miRNA name:** hvu-mir-2911

**Pre-microRNA sequence:** AGACUGAGAGCUCUUUCUUGAUUCUAUGGGUGGUGGUGCAUGGCCGUUCUUAGUUGGUGGAGCGAUUUGUC (71 bases)

**Mature sequence:** UAGUUGGUGGAGCGAUUUGUC (21 bases)

**ΔG**= -19.6

**% GC content**: 49

**MEFI**: **0.56**

**
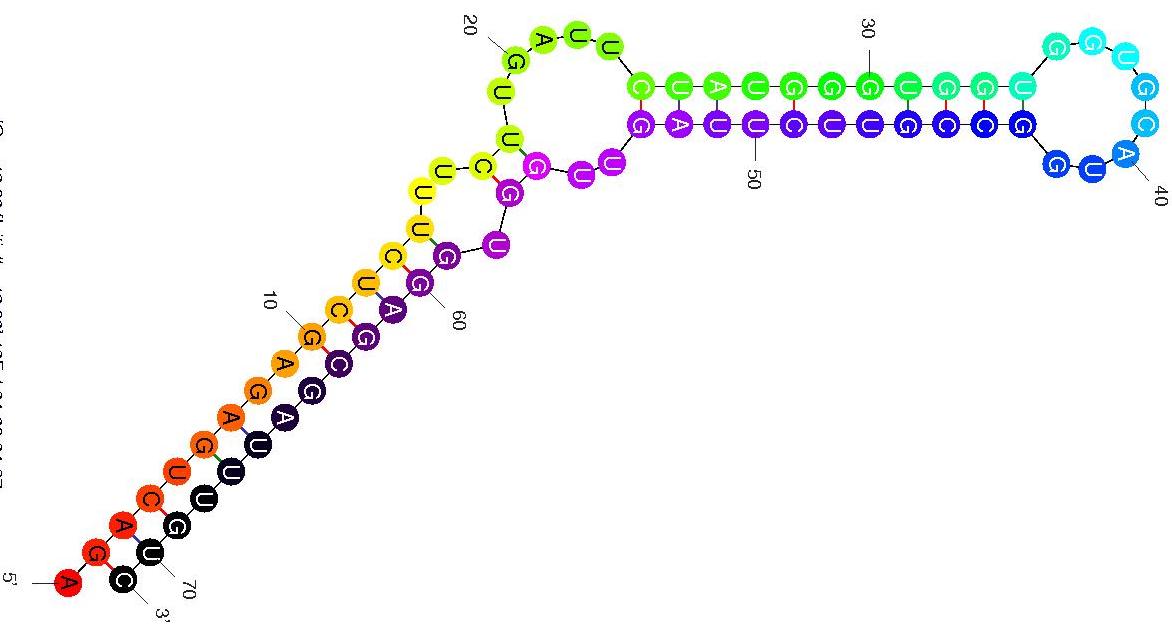
**

**Figure No: 33**

**UnigenCode:** Unigene28333

**miRNA name:** hvu-mir-5066

**Pre-microRNA sequence:** CUGUUGAAGUGUAUAUGUGGAGUGUCUAGCCCUUUUCAUUAGUUCGGACUUUUGGUUGCGUUGACUAGUGCAUGAAGCUC (80 bases)

**Mature sequence:** AAGUGUAUAUGUGGAGUGUCU (21 bases)

**ΔG**= -11.70


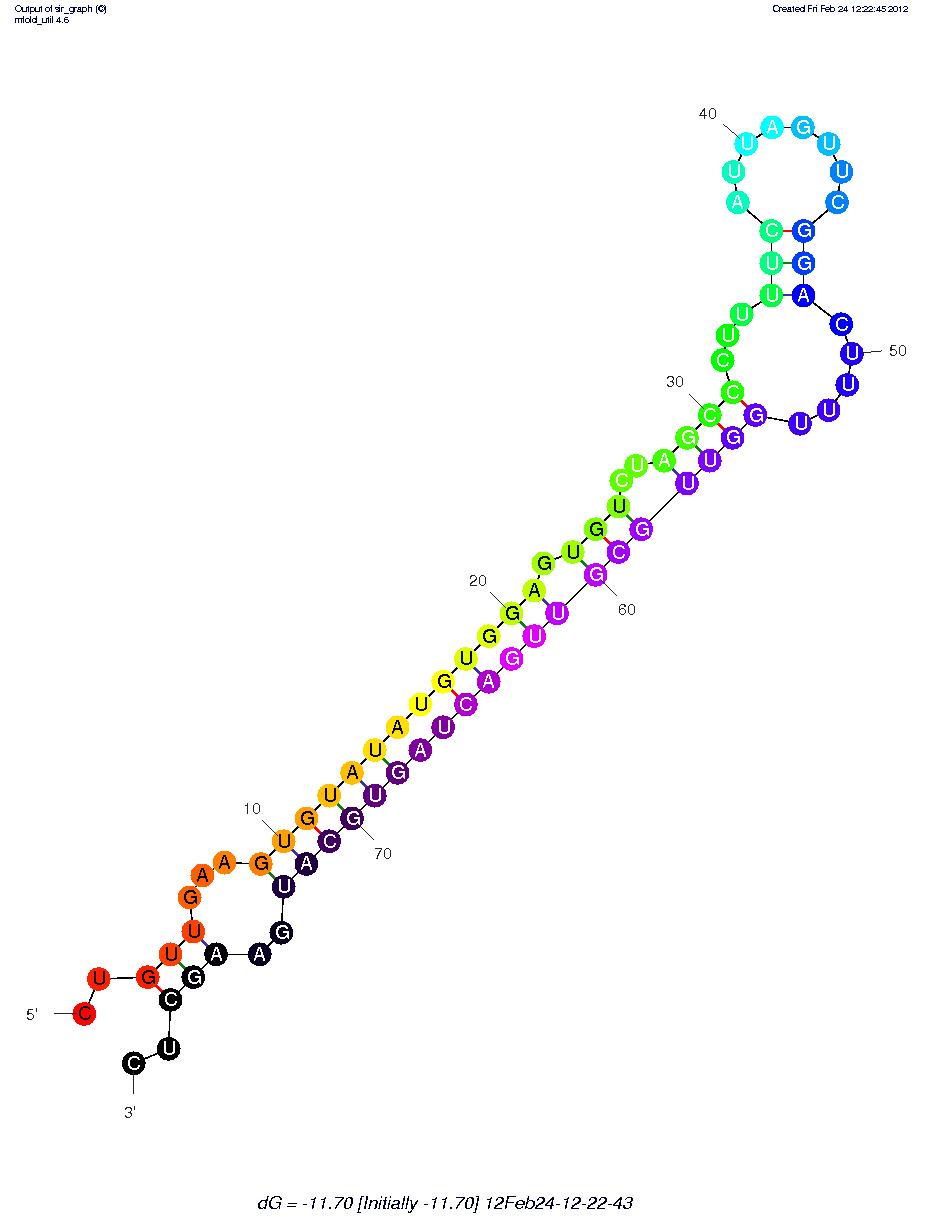
 **% GC content**: 44

**MEFI**: **0.33**

**Figure No: 33**

**UnigenCode:** Unigene32485

**miRNA name:** hvu-mir-166

**Pre-microRNA sequence:** GGAAUCCGGACCAGGCUUCAUUCCCAUCAUUUGCACCCAUUCGACAGCGGUUCCGGUCGCC (61 bases)

**Mature sequence:** CCGGACCAGGCUUCAUUCCCA (21 bases)

ΔG= -12.50

% GC content: 59.0

MEFI: **0.34**


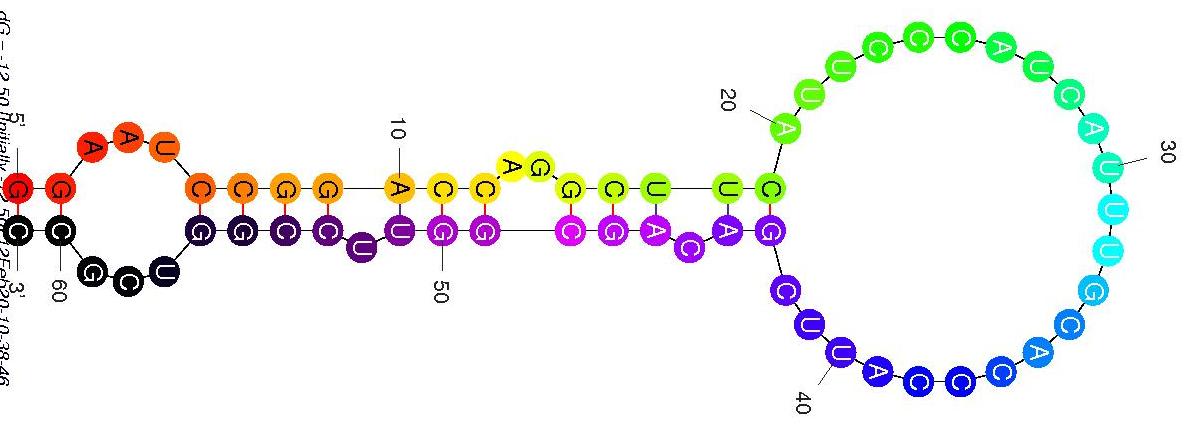


**Figure No: 34**
