# Supplementary material for: Boron Stress Responsive MicroRNAs and Their Targets in Barley
Source: PLoS One. 2013 Mar 26;8(3):e59543. doi: 10.1371/journal.pone.0059543 (PMC3608689; doi:10.1371/journal.pone.0059543)
Supplement: Table S1 — MicroRNA guided cleavage sites by degradome analysis. (DOCX) [file pone.0059543.s002.docx]

**Supplementary Table S1.** MicroRNA guided cleavage sites by degradome analysis

| **miRNAs** | **Target Description** | \| **Query Name** \| **Putative Degradome Cleavage Site** \| **Target Name** \| \| --- \| --- \| --- \| |
| --- | --- | --- | --- | --- | --- |
| hvu-miR156  hvu-miR157 | Squamosa promoter-binding protein | \|  \|  \| \| \| \|  \| \| \| --- \| --- \| --- \| --- \| --- \| --- \| --- \| \| CL13226.Contig1_All \| 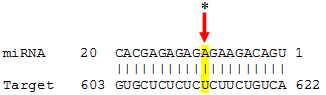   \|  \| \| --- \| \| \| \| \| \| Squamosa promoter-binding-like protein 18 \| \| \| \| \| \| \|  \|  \|  \|  \|  \|  \|  \| \| CL11026.Contig1_All \| 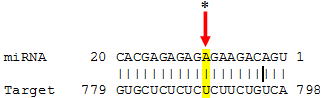   \|  \| \| --- \| \| \| \| \| \| SBP transcription factor \| \| \| \| \| \| \| \|  \|  \|  \|  \|  \|  \|  \| \| CL11193.Contig1_All \| 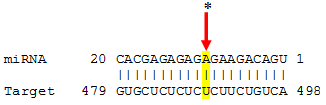   \|  \| \| --- \| \| \| \| \| \| Squamosa promoter-binding-like protein 2 \| \| \| \| \| \| \| \|  \|  \|  \|  \|  \|  \|  \| \| CL38155.Contig1_All \| 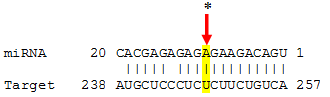   \|  \| \| --- \| \| \| \| \| \| Squamosa promoter-binding-like protein 13 \| \| \| \| \| \| \| |
| hvu-miR159  hvu-miR159a  hvu-miR159b | MYB family transcription factor (GAMyb transcription factor family) | \| CL32877.Contig1_All \| 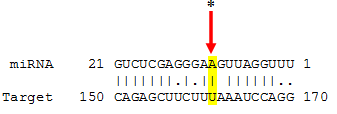   \|  \| \| --- \| \| \| MYB transcription factor family \| \| --- \| --- \| --- \| --- \| \| \| \| \| |
| hvu-miR160 | Auxin response factor (ARF) | \| CL7269.Contig1_All \| 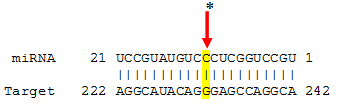   \|  \| \| --- \| \| \| Auxin response factor (ARF) \| \| --- \| --- \| --- \| --- \| \| \| \| \| |
| hvu-miR164a  hvu-miR164b | NAC transcription factor (NAC) | \| 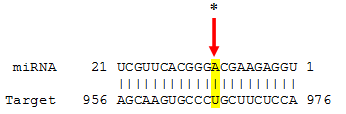      CL6305.Contig2_All \| \|  \| \| --- \| \| \| \| \| \| NAC transcription factor 7 \| \| \| --- \| --- \| --- \| --- \| --- \| --- \| --- \| --- \| \| \| \| \| \|  \|  \|  \|  \|  \|  \|  \| \| 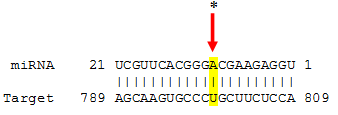  CL1686.Contig1_All \| \|  \| \| --- \| \| \| \| \| \| NAC transcription factor (NAC) \| \| \| \| \| \| |
|  |  | \| Unigene29351_All \| 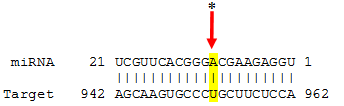   \|  \| \| --- \| \| \| \| \| \| NAC domain protein NAC1 \| \| \| --- \| --- \| --- \| --- \| --- \| --- \| --- \| --- \| \| \| \| \| \| 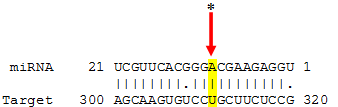 \|  \|  \|  \|  \|  \|  \| \| CL19527.Contig1_All \| \|  \| \| --- \| \| \| \| \| \| NAC domain-containing protein \| \| \| \| \| \| \|  \|  \|  \|  \|  \|  \|  \| \| 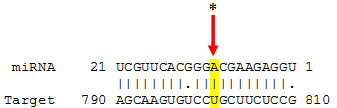  CL3897.Contig1_All \| \|  \| \| --- \| \| \| \| \| \| NAC domain-containing protein \| \| \| \| \| \| \|  \|  \|  \|  \|  \|  \|  \| \| 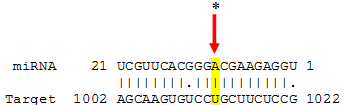  CL8731.Contig1_All \| \|  \| \| --- \| \| \| \| \| \| NAC domain class transcription factor (NAC) \| \| \| \| \| \| |
|  |  | 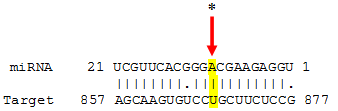   \| Unigene5170_All \| \|  \| \| --- \| \| \| NAC transcription  factor (NAC) \| \| --- \| --- \| --- \| --- \| \| \| \| \| |
| hvu-miR165  hvu-miR166c | Class III Homeodomain-leucine zipper (HD-ZIP III) proteins | \| 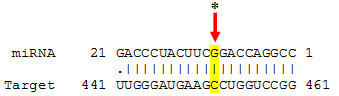  CL153.Contig8_All \| \|  \| \| --- \| \| \| \| \| \| HD-Zip III protein HB8 \| \| \| --- \| --- \| --- \| --- \| --- \| --- \| --- \| --- \| \| \| \| \| \|  \|  \|  \|  \|  \|  \|  \| \| 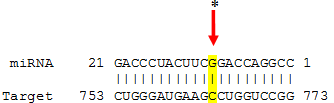  CL153.Contig11_All \| \|  \| \| --- \| \| \| \| \| \| Homeobox-leucine zipper protein HOX9 \| \| \| \| \| \| |
| hvu-miR168a (3p)  hvu-miR168b (3p) | AGO1 (ARGONAUTE 1) | \| CL3360.Contig1_All \| 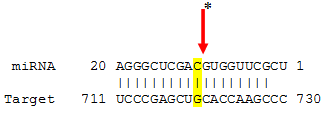   \|  \| \| --- \| \| \| Argonaute  protein (AGO1b) \| \| --- \| --- \| --- \| --- \| \| \| \| \| |
| hvu-miR169 | Nuclear transcription factor Y subunit (NF-Y) | \| 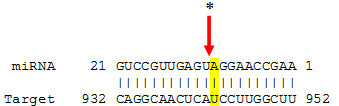  CL5590.Contig1_All \| \|  \| \| --- \| \| \| \| \| \| Nuclear transcription factor Y subunit A-3 (NF-Y) \| \| \| --- \| --- \| --- \| --- \| --- \| --- \| --- \| --- \| \| \| \| \| \| 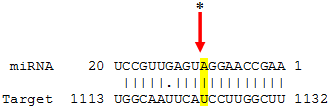 \|  \|  \|  \|  \|  \|  \| \| CL3849.Contig1_All \| \|  \| \| --- \| \| \| \| \| \| Nuclear transcription factor Y subunit A9 (NF-Y) \| \| \| \| \| \| \| 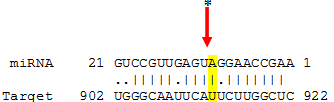 \|  \|  \|  \|  \|  \|  \| \| CL2801.Contig1_All \| \|  \| \| --- \| \| \| \| \| \| Nuclear transcription factor Y subunit A9 (NF-Y) \| \| \| \| \| \| |
| hvu-miR172c  hvu-miR172d | AP-2 Transcription Factors | 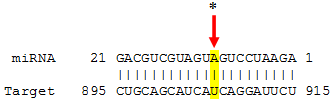   \| CL27047.Contig1_All \| \|  \| \| --- \| \| \| Transcription factor AP2D23-like \| \| --- \| --- \| --- \| --- \| \| \| \| \| |
| hvu-miR319a  hvu-miR319c | MYB transcription factor family | 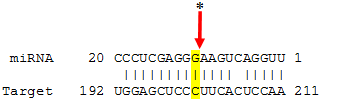   \| CL32877.Contig1_All \| \|  \| \| --- \| \| \| \| \| \| MYB transcription factor family \| \| \| --- \| --- \| --- \| --- \| --- \| --- \| --- \| --- \| \| \| \| \| \| 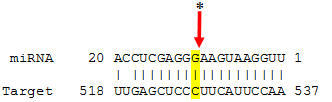 \|  \|  \|  \|  \|  \|  \| \| CL2226.Contig1_All \| \|  \| \| --- \| \| \| \| \| \| Transcription factor AP2D23-like \| \| \| \| \| \| |
| hvu-miR397 | Laccase mRNA | \| 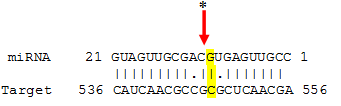CL1278.Contig5_All \| \|  \| \| --- \| \| \| Laccase-4-like \| \| --- \| --- \| --- \| --- \| \| \| \| \| |
| hvu-miR399 | Phosphate transporter 2 (PHO2) or  Putative ubiquitin conjugating enzyme (UBC) | \| CL876.Contig1_All \| 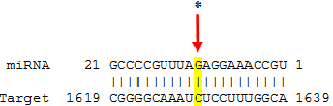   \|  \| \| --- \| \| \| \| \| \| Phosphate transporter 2 (PHO2) or Putative ubiquitin conjugating enzyme (UBC) \| \| \| --- \| --- \| --- \| --- \| --- \| --- \| --- \| --- \| \| \| \| \| \|  \|  \|  \|  \|  \|  \|  \| \|  \|  \| \| \| \|  \| \| \| 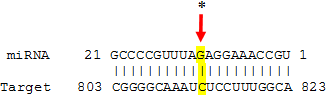  CL876.Contig4_All \| \|  \| \| --- \| \| \| \| \| \| Phosphate transporter 2 (PHO2) or Putative ubiquitin conjugating enzyme (UBC) \| \| \| \| \| \| |
| hvu-miR444  hvu-miR444a  hvu-miR444b  hvu-miR444c | MADS-box transcription factor | 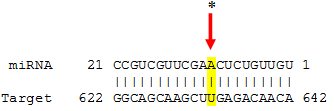   \| CL1260.Contig1_All \| \|  \| \| --- \| \| \| MADS-box  transcription factor \| \| --- \| --- \| --- \| --- \| \| \| \| \| |
|  |  | \| CL3271.Contig2_All \| 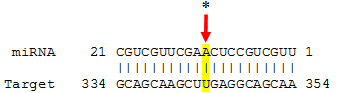   \|  \| \| --- \| \| \| MADS-box  transcription factor \| \| --- \| --- \| --- \| --- \| \| \| \| \| |
| hvu-miR408 | Heterotrimeric G protein alpha subunit or ATPase family gene 1 (AFG1) | 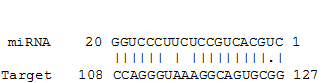   \| CL30341.Contig1_All \| \|  \| \| --- \| \| \| \| \| \| Heterotrimeric G protein alpha subunit \| \| \| --- \| --- \| --- \| --- \| --- \| --- \| --- \| --- \| \| \| \| \| \|  \|  \|  \|  \|  \|  \|  \| \| 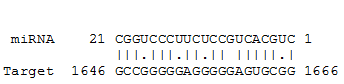Unigene31703_All \| \|  \| \| --- \| \| \| \| \| \| ATPase family gene 1 (AFG1) \| \| \| \| \| \| |
| hvu-miR1120 | COV1-like protein | \| 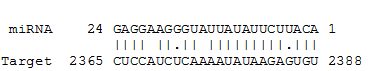  CL58.Contig8_All \| \|  \| \| --- \| \| \| COV1-like protein \| \| --- \| --- \| --- \| --- \| \| \| \| \| |
| hvu-miR1121 | Serine/threonine protein kinase | 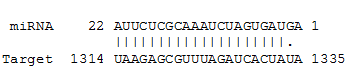   \| CL3697.Contig1_All \| \|  \| \| --- \| \| \| Serine/threonine protein kinase \|  \| \| --- \| --- \| --- \| --- \| --- \| \| \| \| \| \| 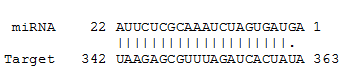Unigene28145_All \| \|  \| \| --- \| \| \| Serine/threonine protein kinase \|  \| \| \| \| \| |
| hvu-miR1122 | Phospholipase A2 or Universal stress protein (USP) or WIR1 | \| 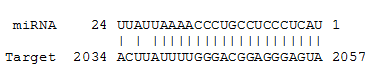  CL1.Contig23_All \| \|  \| \| --- \| \| \| Phospholipase A2 \| \| --- \| --- \| --- \| --- \| \| \| \| \| \| 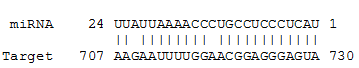  CL2301.Contig1_All \| \|  \| \| --- \| \| \| Universal Stress  Protein (USP) \| \| \| \| \| \| 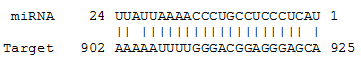 CL2147.Contig2_All \| \|  \| \| --- \| \| \| WIR1 \| \| \| \| \| |
| hvu-miR1126 | Zinc finger ccch domain-containing protein | \| 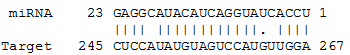CL6067.Contig1_All CL6067.Contig3_All \| \|  \| \| --- \| \| \| \| \| \| Zinc finger ccch domain-containing protein \| \| \| --- \| --- \| --- \| --- \| --- \| --- \| --- \| --- \| \| \| \| \| \|  \|  \|  \|  \|  \|  \|  \| \| 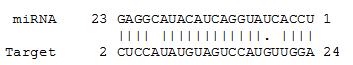CL6067.Contig2_All \| \|  \| \| --- \| \| \| \| \| \| Zinc finger ccch domain-containing protein \| \| \| \| \| \| |
| hvu-miR2004 | PHD finger family protein or AP-1 complex subunit or Subtilase family protein or Tetratricopeptide repeat-containing protein or Transcription elongation factor (TFIIS) family protein | \| 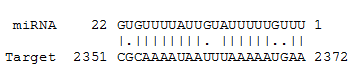CL1242.Contig3_All \| \|  \| \| --- \| \| \| \| \| \| PHD finger family protein \| \| \| --- \| --- \| --- \| --- \| --- \| --- \| --- \| --- \| \| \| \| \| \|  \|  \|  \|  \|  \|  \|  \| \| 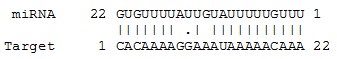CL6239.Contig1_All \| \|  \| \| --- \| \| \| \| \| \| AP-1 complex subunit \| \| \| \| \| \| \|  \|  \|  \|  \|  \|  \|  \| \| 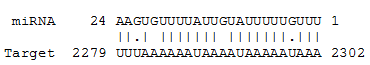CL904.Contig1_All \| \|  \| \| --- \| \| \| \| \| \| Subtilase family protein \| \| \| \| \| \| |
|  |  | \| 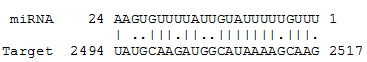CL162.Contig5_All \| \|  \| \| --- \| \| \| \| \| \| Tetratricopeptide repeat-containing protein \| \| \| --- \| --- \| --- \| --- \| --- \| --- \| --- \| --- \| \| \| \| \| \|  \|  \|  \|  \|  \|  \|  \| \| CL17869.Contig1_All \| 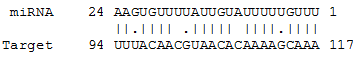   \|  \| \| --- \| \| \| \| \| \| Transcription elongation factor (TFIIS) family protein \| \| \| \| \| \| |
| hvu-miR2007 | Protein phosphatase or Serine/arginine repetitive matrix protein | \| CL2929.Contig1_All \| 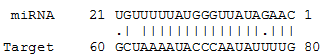   \|  \| \| --- \| \| \| \| \| \| Protein  phosphatase 2C \| \| \| --- \| --- \| --- \| --- \| --- \| --- \| --- \| --- \| \| \| \| \| \|  \|  \|  \|  \|  \|  \|  \| \| CL6012.Contig1_All \| 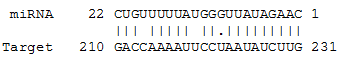   \|  \| \| --- \| \| \| \| \| \| Serine/arginine repetitive matrix protein 1 \| \| \| \| \| \| |
| hvu-miR2014 | Phospholipid-translocating ATPase or GTP-binding protein or Ethylene responsive factor or Transcription factor jumonji | \| CL283.Contig1_All \| 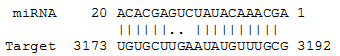   \|  \| \| --- \| \| \| Phospholipid-translocating ATPase \| \| --- \| --- \| --- \| --- \| \| \| \| \| |
|  |  | \| CL7041.Contig1_All \| 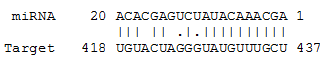   \|  \| \| --- \| \| \| \| \| \| GTP-binding protein \| \| \| --- \| --- \| --- \| --- \| --- \| --- \| --- \| --- \| \| \| \| \| \|  \|  \|  \|  \|  \|  \|  \| \| CL2423.Contig1_All \| 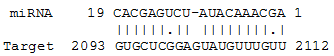   \|  \| \| --- \| \| \| \| \| \| Ethylene responsive factor \| \| \| \| \| \| \|  \|  \|  \|  \|  \|  \|  \| \| CL3225.Contig1_All \| 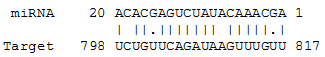   \|  \| \| --- \| \| \| \| \| \| Transcription factor jumonji \| \| \| \| \| \| |
| hvu-miR2019 | Tubulin-tyrosine ligase family | \| CL326.Contig1_All \| 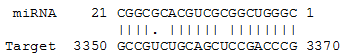   \|  \| \| --- \| \| \| Tubulin-tyrosine  ligase family \| \| --- \| --- \| --- \| --- \| \| \| \| \| |
| hvu-miR2021 | Rough sheath 2-interacting KH domain protein (RIK) or Lysophosphatidylcholine Acyltransferase or Respiratory burst oxidase-like protein F2 or Cytochrome P450 | \| CL527.Contig3_All \| 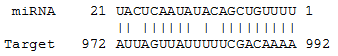   \|  \| \| --- \| \| \| Rough sheath  2-interacting  KH domain  protein (RIK) \| \| --- \| --- \| --- \| --- \| \| \| \| \| |
|  |  | \| 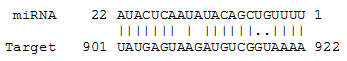Unigene27511_All \| \|  \| \| --- \| \| \| \| \| \| Lysophosphatidylcholine Acyltransferase \| \| \| --- \| --- \| --- \| --- \| --- \| --- \| --- \| --- \| \| \| \| \| \|  \|  \|  \|  \|  \|  \|  \| \| CL318.Contig4_All \| 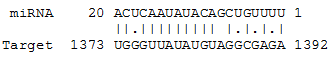   \|  \| \| --- \| \| \| \| \| \| Respiratory burst oxidase-like protein F2 \| \| \| \| \| \| \|  \|  \|  \|  \|  \|  \|  \| \| CL2680.Contig1_All \| 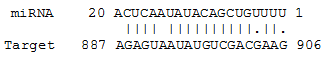   \|  \| \| --- \| \| \| \| \| \| Cytochrome P450 \| \| \| \| \| \| |
| hvu-miR2024a | MADS box protein-like protein or Zinc finger family protein | \| CL3271.Contig2_All \| 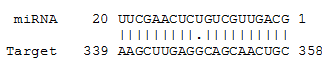   \|  \| \| --- \| \| \| \| \| \| MADS box protein-like protein \| \| \| --- \| --- \| --- \| --- \| --- \| --- \| --- \| --- \| \| \| \| \| \|  \|  \|  \|  \|  \|  \|  \| \| CL9100.Contig1_All \| 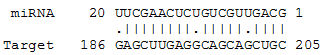   \|  \| \| --- \| \| \| \| \| \| Zinc finger family protein \| \| \| \| \| \| |
|  |  | \| CL40097.Contig1_All \| 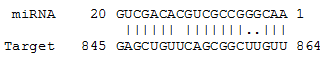   \|  \| \| --- \| \| \| \| \| \| (E)-beta-caryophyllene/beta-elemene synthase \| \| \| --- \| --- \| --- \| --- \| --- \| --- \| --- \| --- \| \| \| \| \| \|  \|  \|  \|  \|  \|  \|  \| \| Unigene30593_All \| 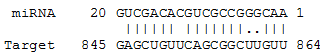   \|  \| \| --- \| \| \| \| \| \| (E)-beta-caryophyllene/beta-elemene synthase \| \| \| \| \| \| |
| hvu-miR2910 | Glycine rich protein 3 or Glyceraldehyde-3-phosphate dehydrogenase, cytosoli or Phosphatidylinositol-4-phosphate 5-kinase 9 or Ubiquitin-associated protein | \| CL40314.Contig1_All \| 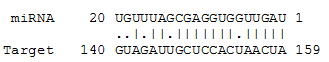   \|  \| \| --- \| \| \| \| \| \| glycine rich protein 3 \| \| \| --- \| --- \| --- \| --- \| --- \| --- \| --- \| --- \| \| \| \| \| \|  \|  \|  \|  \|  \|  \|  \| \| 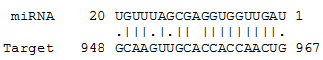CL386.Contig2_All \| \|  \| \| --- \| \| \| \| \| \| glyceraldehyde-3- phosphate dehydrogenase,  cytosoli \| \| \| \| \| \| \| Unigene11586_All \| 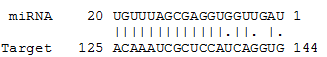   \|  \| \| --- \| \| \| \| \| \| Phosphatidylinositol  l-4-phosphate 5-kinase 9 \| \| \| \| \| \| |
|  |  | \| 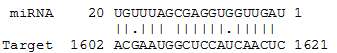  CL5067.Contig2_All \| \|  \| \| --- \| \| \| ubiquitin-  associated protein \| \| --- \| --- \| --- \| --- \| \| \| \| \| |
| hvu-miR2911 | ASF/SF2-like pre-mRNA splicing factor SRP32 or Hydroxyproline-rich glycoprotein family protein | \| CL17424.Contig1_All \| 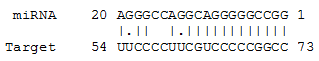   \|  \| \| --- \| \| \| \| \| \| ASF/SF2-like  pre-mRNA splicing factor SRP32 \| \| \| --- \| --- \| --- \| --- \| --- \| --- \| --- \| --- \| \| \| \| \| \|  \|  \|  \|  \|  \|  \|  \| \| 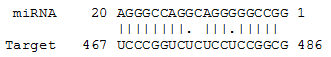CL23524.Contig1_All \| \|  \| \| --- \| \| \| \| \| \| hydroxyproline-rich glycoprotein family protein \| \| \| \| \| \| |
| hvu-miR2914  hvu-miR2916 | Senescence-associated protein or CBL-interacting protein kinase 21 | \| CL8337.Contig1_All \| 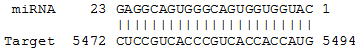   \|  \| \| --- \| \| \| \| \| \| senescence-  associated protein \| \| \| --- \| --- \| --- \| --- \| --- \| --- \| --- \| --- \| \| \| \| \| \|  \|  \|  \|  \|  \|  \|  \| \| 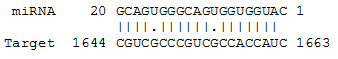CL660.Contig7_All \| \|  \| \| --- \| \| \| \| \| \| CBL-interacting  protein kinase 21 \| \| \| \| \| \| |
|  |  | \| 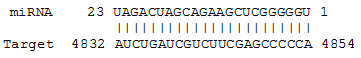 CL8337.Contig1_All \| \|  \| \| --- \| \| \| senescence-  associated protein \| \| --- \| --- \| --- \| --- \| \| \| \| \| |
| hvu-miR5048 | RPG1 or Serine/threonine protein kinase or NAC domain-containing protein 18 or Serine/threonine kinase-like protein | \| CL26250.Contig1_All \| 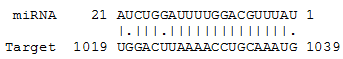   \|  \| \| --- \| \| \| \| \| \| RPG1 \| \| \| --- \| --- \| --- \| --- \| --- \| --- \| --- \| --- \| \| \| \| \| \|  \|  \|  \|  \|  \|  \|  \| \| 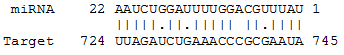CL2067.Contig1_All \| \|  \| \| --- \| \| \| \| \| \| Serine/threonine protein kinase \| \| \| \| \| \| \|  \|  \|  \|  \|  \|  \|  \| \| CL5978.Contig2_All \| 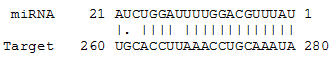   \|  \| \| --- \| \| \| \| \| \| NAC domain-  containing  protein 18 \| \| \| \| \| \| \|  \|  \|  \|  \|  \|  \|  \| \| 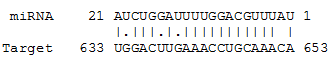CL421.Contig2_All \| \|  \| \| --- \| \| \| \| \| \| Serine/threonine kinase-like protein \| \| \| \| \| \| |
| hvu-miR5049 | Tubby protein-like | \| 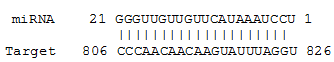CL9685.Contig1_All \| \|  \| \| --- \| \| \| Tubby  protein-like \| \| --- \| --- \| --- \| --- \| \| \| \| \| |
| hvu-miR5052 | Cyclophilin | \| CL27515.Contig1_All \| 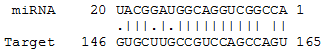   \|  \| \| --- \| \| \| Cyclophilin \| \| --- \| --- \| --- \| --- \| \| \| \| \| |
| hvu-miR5053 | Chlorophyll a/b-binding protein or Predicted protein | \| CL40448.Contig1_All \| 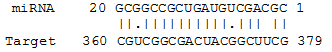   \|  \| \| --- \| \| \| \| \| \| Chlorophyll  a/b-binding protein \| \| \| --- \| --- \| --- \| --- \| --- \| --- \| --- \| --- \| \| \| \| \| \|  \|  \|  \|  \|  \|  \|  \| \| CL33769.Contig1_All \| 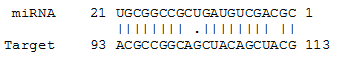   \|  \| \| --- \| \| \| \| \| \| Predicted protein \| \| \| \| \| \| |
| hvu-miR5056 | RNA polymerase beta subunit | \| CL179.Contig1_All \| 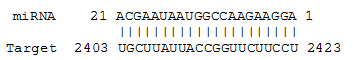   \|  \| \| --- \| \| \| RNA polymerase  betasubunit \| \| --- \| --- \| --- \| --- \| \| \| \| \| |
| hvu-miR5066 | Carbohydrate transporter/ sugar porter/ transporter or Serine/threonine protein kinase | \| CL21592.Contig1_All \| 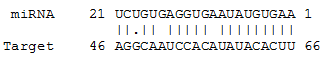   \|  \| \| --- \| \| \| \| \| \| Carbohydrate transporter/ sugar porter/ transporter \| \| \| --- \| --- \| --- \| --- \| --- \| --- \| --- \| --- \| \| \| \| \| \|  \|  \|  \|  \|  \|  \|  \| \| CL6.Contig12_All \| 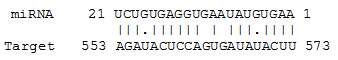   \|  \| \| --- \| \| \| \| \| \| serine/  threonine  protein kinase \| \| \| \| \| \| |
