# Supplementary material for: Boron Stress Responsive MicroRNAs and Their Targets in Barley
Source: PLoS One. 2013 Mar 26;8(3):e59543. doi: 10.1371/journal.pone.0059543 (PMC3608689; doi:10.1371/journal.pone.0059543)
Supplement: Table S2 — Primers used for miRNA validation and measurement detected in this study. (DOCX) [file pone.0059543.s003.docx]

| Hvu-miRNAs names | RT Primers  (5'->3') | Forward Primers(F)  (5'->3') | Universal Reverse Primer(R)  (5'->3') |
| --- | --- | --- | --- |
| Hvu-miR156 | 5'-GTCGTATCCAGTGCAGGGTCCGAGGTATTCGCACTGGATACGACGTGCTC-3' | 5'- TCGCGTGACAGAAGAGAGA-3' | 5'-GTGCAGGGTCCGAGGT-3' |
| Hvu-miR159 | 5’-GTCGTATCCAGTGCAGGGTCCGAGGTATTCGCACTGG-3’ | 5’-GCGGCGGTATTGGAGTGAAGGGA-3’ |  |
| Hvu-miR164 | 5’-GTCGTATCCAGTGCAGGGTCCGAGGTATTCGCACTGG-3’ | 5'- TCGCTTGGAGAAGCAGGGCA-3’ |  |
| Hvu-miR166 | 5’- GTCGTATCCAGTGCAGGGTCCGAGGTATTCGCACTGG-3’ | 5'- GCGGCGGTCGGACCAGGCTTCAT-3’ |  |
| Hvu-miR 171 | 5’-GTCGTATCCAGTGCAGGGTCCGAGGTATTCGCACTGG-3’ | 5'-TTCCTTATTGAGCCGTGCC -3’ |  |
| Hvu-miR168 | 5'-GTCGTATCCAGTGCAGGGTCCGAGGTATTCGCACTGGATACGACATTCAC-3' | 5'- TTCCTTGATCCCGCCTTGCACCAA-3' |  |
| Hvu-miR 395 | 5'-GTCGTATCCAGTGCAGGGTCCGAGGTATTCGCACTGG-3' | 5'- CGGCGGCTGAAGTGTTTGGGGG-3' |  |
| Hvu-miR 396 | 5'GTCGTATCCAGTGCAGGGTCCGAGGTATTCGCACTGGATACGACAGTTCA-3' | 5'-TCGCGTTCCACAGCTTTCT -3' |  |
| Hvu-miR 414 | 5'-GTCGTATCCAGTGCAGGGTCCGAGGTATTCGCACTGGATACGACGACGAT-3' | 5'-CGGCGGTCATCTTCATCATC -3’ |  |
| Hvu-miR 1120 | 5'-GTCGTATCCAGTGCAGGGTCCGAGGTATTCGCACTGGATACGACCTCCGT-3' | 5'- TCGCGACATTCTTATATTATGGG-3' |  |
| Hvu-miR 5048 | 5'-GTCGTATCCAGTGCAGGGTCCGAGGTATTCGCACTGGATACGACTTAGAC-3' | 5'- TCGCTTATTTGCAGGTTTTAG-3' |  |
