# Supplementary material for: Boron Stress Responsive MicroRNAs and Their Targets in Barley
Source: PLoS One. 2013 Mar 26;8(3):e59543. doi: 10.1371/journal.pone.0059543 (PMC3608689; doi:10.1371/journal.pone.0059543)
Supplement: Table S3 — Primers used for target mRNA validation and measurement detected in this study. (DOCX) [file pone.0059543.s004.docx]

| Hvu-Tar-miRNAs names | Target genes | Product size | Forward Primers(F)  (5'->3') | Reverse Primers(R)  (5'->3') |
| --- | --- | --- | --- | --- |
| Hvu-Tar-miR156 | Squamosa promoter-binding protein like 11  (SPL11)-Predicted protein | 89 | 5’- CGTCGTAAGCCACAAGGAG-3’ | 5’- GACACCCCATTCCACAACA-3’ |
| Hvu-Tar-miR159 | putative boron transporter 2 | 96 | 5’- GCTTATGGTGATCGTGTGGA-3’ | 5’- TCCCAAGGAAGTGGACTGAA-3’ |
| Hvu-Tar-miR164-miR396-miR414 | Mitochondrial alternative oxidase 1 (AOX1) | 116 | 5’-CGTCCAGGGCGTTTTCTT-3’ | 5’-GGAACTCGGTGTAGGAGTGG-3’ |
| Hvu-Tar-miR166 | phenylalanine ammonia-lyase | 120 | 5’-CTTCTGCGAGGTGATGAATG-3’ | 5’-GAGCTGCCTTCAAGAATGTG-3’ |
| Hvu-Tar-mir168 | AGO1 | 120 | 5’-CGCCCGATAGATCGAGAAA-3’ | 5’-AGCCTCAACAGAAGCCAGAG-3’ |
| Hvu-Tar-mir171 | predicted protein | 110 | 5’-CAGCTGTTGTCACAGGGAAG-3’ | 5’-TCCGTATTCAGCCAGGAGAG-3’ |
| Hvu-Tar-mir395 | sulfate adenylyltransferase 4 | 229 | 5'-CAAAACGTTCAATACCCATTTCTC-3 | 5’-CAAAACGTTCAATACCCATTTCTC-3’ |
| Hvu-Tar-mir1120 | 4,5-DOPA dioxygenase extradiol-like protein (AT4G15093) | 115 | 5’- GGGACACCAAATTCCCATCT-3’ | 5’- TCAATTGCTCCTGGTGCTTC-3’ |
| Hvu-Tar-mir5048 | serine/threonine kinase-like protein ABC1037 | 88 | 5’- TATGCCGTCCCATGTATCTC-3’ | 5’- TATTGTAGGCCTTCGGGTTC-3’ |
